# Sex pheromone biosynthetic pathways are conserved between moths and the butterfly *Bicyclus anynana*

**DOI:** 10.1038/ncomms4957

**Published:** 2014-05-27

**Authors:** Marjorie A Liénard, Hong-Lei Wang, Jean-Marc Lassance, Christer Löfstedt

**Affiliations:** 1 Department of Biology; Pheromone Group, Lund University, Lund SE-22362, Sweden; 2 Department of Organismic and Evolutionary Biology, Harvard University, Cambridge, Massachusetts 02138, USA; 3 These authors contributed equally to this work

## Abstract

Although phylogenetically nested within the moths, butterflies have diverged extensively in a number of life history traits. Whereas moths rely greatly on chemical signals, visual advertisement is the hallmark of mate finding in butterflies. In the context of courtship, however, male chemical signals are widespread in both groups although they likely have multiple evolutionary origins. Here, we report that in males of the butterfly *Bicyclus anynana*, courtship scents are produced *de novo* via biosynthetic pathways shared with females of many moth species. We show that two of the pheromone components that play a major role in mate choice, namely the (*Z*)-9-tetradecenol and hexadecanal, are produced through the activity of a fatty acyl Δ11-desaturase and two specialized alcohol-forming fatty acyl reductases. Our study provides the first evidence of conservation and sharing of ancestral genetic modules for the production of FA-derived pheromones over a long evolutionary timeframe thereby reconciling mate communication in moths and butterflies.

## Abstract (web summary)

Little is known about the evolutionary origins of the genes involved in butterfly pheromone synthesis. Here, Liénard *et al.* show that the biosynthetic pathways involved in the production of male courtship scents of the butterfly, *Bicyclus anynana*, are shared with females of many moth species.

## Graphical abstract

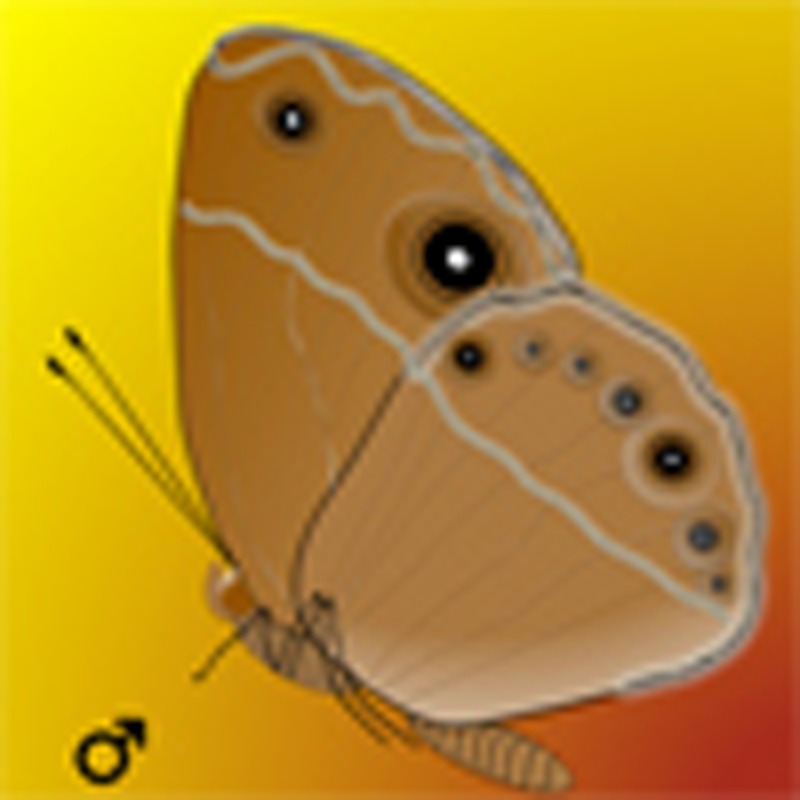

The order Lepidoptera comprises an estimated 160,000 species^1^ and is thought to have arisen in concert with flowering plants about 140 Myr ago^2^. Traditionally, it has been divided into two major subgroups comprising the moths (suborder Heterocera) and butterflies (suborder Rhopalocera)^3^, the latter having diverged from their common moth ancestors in the Early Cretaceous about 110 to 100 Myr ago^2^,^4^, with subsequent lineage-specific bursts of radiations occurring about 70 Myr ago^2^. The *ca* 18,000 described species of extant butterflies (Papilionoidea and Hesperiidae)^4^ have diverged extensively from their moth relatives in a number of life history traits including among others a diurnal lifestyle, bright appearances, body structures, antennal shapes and mate-finding behaviours^5^,^6^,^7^.

The switch from nocturnal to diurnal behaviour and the corresponding increased dependence on visual communication for food and mate finding have led to the widespread assumption that this group of insects has undergone a global decrease in their olfactory capabilities. Indeed, female butterflies seem to have lost the long-distance pheromones on which their moth relatives rely almost exclusively, and mate finding is typically performed by patrolling males in stereotyped and visually oriented search behaviours^8^. Nevertheless, recent studies on the genomic architecture of the olfactory protein and receptor repertoires^9^, the neuroanatomy^10^,^11^ and the behavioural responses to plant and conspecific odours^12^,^13^ have begun to reveal remarkable commonalities in the olfactory system of butterflies and their nocturnal cousins, the moths.

Also, chemical signalling in the form of scent bouquets disseminated by courting males at close range has long been known to complement visual patterns in many butterfly species^8^, which may play a decisive role in species recognition and female mate choice^5^,^14^. The extant butterfly diversity is paralleled by a great chemical diversity in volatile male pheromone components, which include alkaloid derivatives, terpenoids, aromatics or carboxylic acids that are in many cases suggested or proven to be plant derived^5^,^7^,^12^,^14^,^15^,^16^. The great variety in male scent suggests, however, that the butterfly signalling traits and underlying biosynthetic machineries have evolved multiple times independently during the course of evolution^17^. Scent-releasing structures can be found on virtually any part of the body (for example, abdominal hair pencils, wing androconia and brushes), which argues further in favour of the multiple origins of the associated odours^6^.

*Bicyclus anynana* (Nymphalidae) males produce a pheromone blend comprising a phytol-derived compound, the (2*R*, 6*R*, 10*R*)-6,10,14 trimethylpentadecan-2-ol together with the fatty acid (FA) derivatives (*Z*)-9-tetradecenol (Z9-14:OH) and hexadecanal (16:Ald)^13^,^18^. This pheromone bouquet is released during a ritualized courtship display from two sets of modified scale structures—the so-called *androconia*—located on the forewings and hindwings^18^,^19^. Two of the male pheromone components, namely Z9-14:OH and 16:Ald, are structurally identical to many known female moth sex pheromone components^20^,^21^; however, their origin remains uncertain due to the multiple alternative routes possibly leading to these chemical structures.

The double bond in Δ9 position is a widespread structural feature in nature owing to the presence of integral metabolic membrane fatty acyl-CoA Δ9-desaturases^22^. In insects, Δ9-desaturases serve in biosynthetic pathways towards cuticular hydrocarbons in the fruit fly *Drosophila melanogaster*^23^,^24^, scent-marking pheromones in male bumblebees^25^ and long-range sex pheromones in some female moths^26^,^27^. However, in the latter group, molecular reconstructions of biosynthetic pathways revealed that the majority of chemical structures derive from the evolution of novel desaturase multigene families absent from other insect orders^27^,^28^. Specifically, Δ11-desaturases form a key gene lineage^29^,^30^,^31^, of which orthologues encode diverse functional classes with particular regiospecificities (for example, Δ6 (ref. 32), Δ10 (ref. 33), Δ11 (refs 28, 31), bifunctional Δ10-12 (refs 34, 35) and Δ11-13 (ref. 36)). When acting in concert with chain-shortening enzymes, desaturases take part in the production of highly diverse unsaturated FA structures, precursors of the large repertoire of moth pheromones, including Δ9-isomers^29^,^30^. Owing to the fact that the Δ11-desaturase lineage arose in Lepidoptera before the divergence of the butterflies and moths more than 120 Myr ago^28^, butterfly pheromone production likely evolved under a scenario involving either the evolutionary conservation and specialization of moth-like Δ11-desaturases to serve male reproductive functions or the loss of Δ11-desaturases in connection with the disappearance of female pheromones in butterflies followed by recruitment of a Δ9-desaturase for male pheromone production.

Alcohol-producing fatty acyl-CoA reductases (FARs) catalyse the reduction of FAs to fatty alcohols and are also found widely in nature from plants^37^,^38^, invertebrates^39^,^40^,^41^ including moths^42^,^43^,^44^,^45^,^46^, to birds^47^ and mammals^48^. There is a considerable diversity of FAR genes underlying similar functions and activity profiles in spite of phylogenetic unrelatedness. This is notably illustrated by FARs involved in wax ester biosynthetic pathways in a copepod^39^, several hymenopteran^40^,^41^ and bird^47^ species and FARs involved in pheromone pathways in moth species^43^,^46^; all these FARs use the ubiquitous palmitic acid (16:acid) to produce hexadecanol (16:OH), without being necessarily encoded by orthologous genes. By analogy to female moth biosynthesis, the presence of alcohol and aldehyde (the latter presumably being derivative of 16:OH) as functional groups in *B. anynana* male compounds suggests that reduction must occur; yet the involved molecular players could have distinct origins compared with the genes involved in female moth pheromone production.

Due to its unusual pheromone composition *Bicyclus* allows exploration of the evolutionary origins of the genes involved in shaping the biosynthetic modules in butterfly pheromone pathways. Specifically, we dissect the genetic and functional organization of the two major biosynthetic steps—the desaturation and the reduction steps—and using a multidisciplinary approach, we show that *Bicyclus* wing pheromone signalling shares its genetic basis and functional mechanism with those of moths. In addition, we explore other butterfly genomes and provide evidence for the conservation of biosynthetic gene clades in important butterfly systems such as *Danaus* and *Heliconius*. Altogether, our findings bring the first critical evidence to reconcile the evolutionary history of genes that are involved in the production of volatile chemicals in *Bicyclus*, with appropriate tools to start investigating the molecular underpinnings of FA secretions^49^, utilized within the butterflies.

## Results

### The *Bicyclus* pheromone pathway follows moth biosynthesis

Our first aim was to experimentally test putative biosynthetic routes that could lead to the production of adult male pheromone components in *B. anynana,* the Z9-14:OH and 16:Ald, two compounds likely produced *de novo* as part of the insect FA metabolism. The third presumptive pheromone component, trimethylpentadecan-2-ol, was not part of the study as it is a phytol-derived compound^50^ expected to originate from the diet and not related to FA biochemistry.

**Figure 1:**
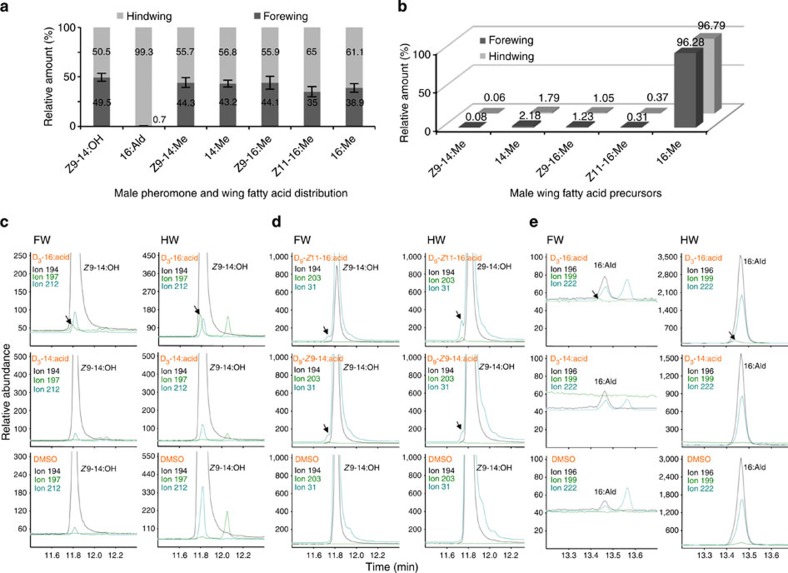
*Bicyclus* male wing pheromone: FA precursors and *in vivo* labelling. (**a**) Wing FA composition in 4-day-old males *B. anynana*. Distribution of the FA-derived pheromone components (Z9-14:OH and 16:Ald) (*n*=10) and the potential immediate FA biosynthetic precursors (*n*=5) in forewings and hindwings. Bars represent s.e.m. Hexadecanal is almost exclusively present in hindwings, whereas the Z9-tetradecanol distributes equally across the two wings^18^. The identified FA precursors distribute across both wings. (**b**) Proportions of FA precursors in each wing relative to the total wing FA content (100%). (**c**–**e**) Analysis of pheromone production in male butterfly wings via biochemical *in vivo* labelling. Panels represent typical gas chromatograms from SIM, which records diagnostic ions according to the number of deuterium atoms in the precursor applied onto the wing androconia (FW: forewing, left columns; HW: hindwing, right columns) and shows incorporation of labelled FA precursors into pheromone components (16:Ald and *Z*9-14:OH). (**c**,**d**) The Z9-14:OH pheromone pathway. The native Z9-14:OH was monitored via its molecular ion at *m/z* 212 and the diagnostic ions at *m/z* 194 [M-18]^+^ or 31 [CH_2_OH]^+^. The upper panels show deuterium incorporation in FW and HW androconia from D_3_-16:acid (**c**) and D_9_-Z11-16:acid (**d**) into Z9-14:OH, which was monitored by ions showing 3 or 9 a.m.u. more, respectively, than the native ion at *m/z* 194. Middle and lower panels display the corresponding chromatogram traces from negative labelling with D_3_-14:acid and DMSO controls in each wing, respectively. (**e**) The 16:Ald pheromone pathway. The native 16:Ald was monitored by the diagnostic ions at *m/z* 222 [M-18]^+^ and 196 [M-44]^+^. The upper panels show incorporation in FW and HW androconia of the labelled D_3_-16:acid into 16:Ald, monitored by a diagnostic ion showing 3 a.m.u. more than the native ion at *m/z* 196, whereas there is no deuterium incorporation when solutions containing D_3_-14:acid or DMSO only are applied onto the wings.

First, we prepared wing extracts from 4-day-old male *B. anynana* individuals. The first male-specific component, *Z*9-14:OH is equally distributed in the forewing and the hindwing^18^ (Fig. 1a). The second male component, 16:Ald is found almost exclusively in the hindwing, more specifically in the androconia (Fig. 1a). We confirmed that its immediate precursor, 16:OH, is undetectable, which is most likely the consequence of its rapid conversion into the final aldehyde form.

**Figure 2:**
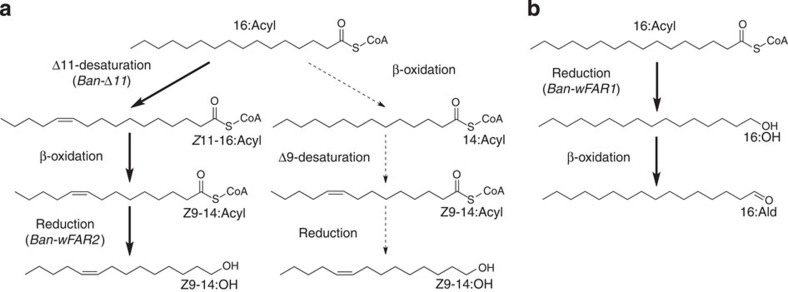
Biosynthetic pathways towards the *B. anynana* male pheromone. Proposed pathways towards the male courtship compounds *Z*9-14:OH (**a**) and 16:Ald (**b**) in *B. anynana*. Alternative pathways are represented based on FAs identified in the male wings. The active pheromone production enzymes characterized in this study (Ban-Δ11, Ban-wFAR1 and Ban-wFAR2) support the reactions indicated with plain arrows.

In order to reveal FA that could represent potential pheromone precursors, we subjected the fore- and hindwing tissues to total lipid extraction. Both forewings and hindwings contained myristic acid (14:acid) and palmitic acid (16:acid), as well as the monounsaturated (*Z*)-9-tetradecenoic acid (*Z*9-14:acid), (*Z*)-9-hexadecenoic acid (*Z*9-16:acid) and (*Z*)-11-hexadecenoic acid (*Z*11-16:acid). The high abundance of palmitic acid, which represented over 96% of the C14-C16 fatty acyl components isolated from the wings (Fig. 1b), together with the presence of unsaturated Z9-14:acid and Z11-16:acid, are in agreement with the postulated biosynthesis of the alcohol and aldehyde components from palmitic acid in *B. anynana* (Fig. 2).

To validate the alternative biosynthetic routes, we carried out topical application of deuterium-labelled C16 and C14 precursors on the respective fore- and hindwing androconial areas. We applied D_3_-16:acid, D_3_-14:acid, D_9_-*Z*11-16:acid and D_9_-*Z*9-14:acid as single-compound solutions onto the androconial region of each wing. Our analyses of wing extracts revealed that deuterium atoms from D_3_-16:acid, D_9_-*Z*11-16:acid and D_9_-*Z*9-14:acid were incorporated into *Z*9-14:OH in both fore- and hindwings (Fig. 1c,d, Supplementary Fig. 1). In contrast, there was no label incorporation from D_3_-14:acid into Z9-14:OH (Fig. 1c,d). Deuterium atoms from D_3_-16:acid were incorporated into the 16:Ald in hindwings (Fig. 1e), whereas diagnostic ions indicative of label incorporation were absent in DMSO controls in all analyses (Fig. 1c–e).

### Butterfly genome mining for FAD and FAR gene orthologues

**Figure 3:**
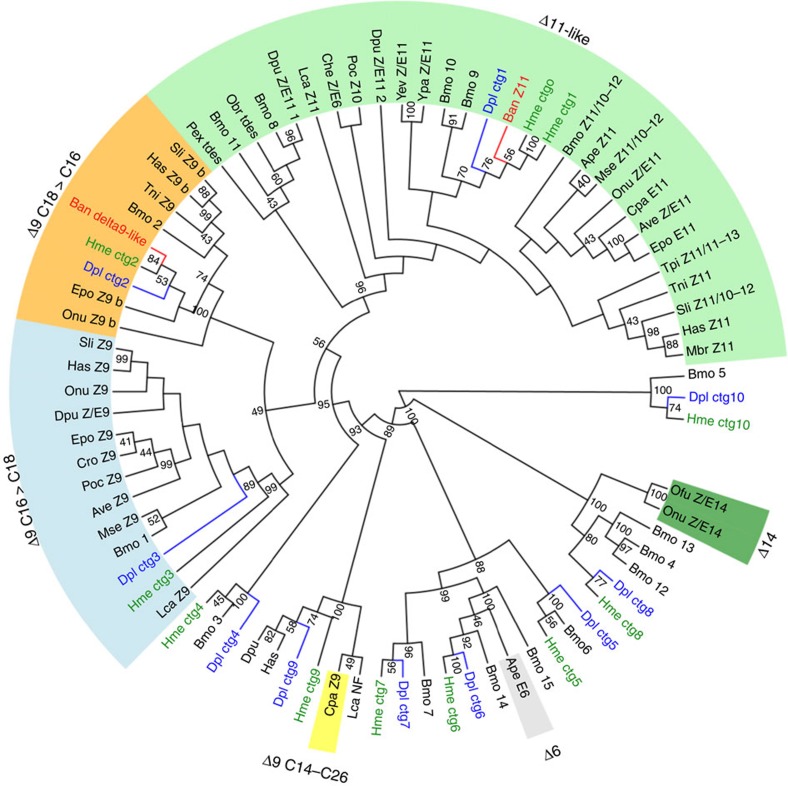
Phylogeny of moths and butterflies FADs. A representative set of moth and butterfly desaturases were used to reconstruct the maximum-likelihood phylogeny. The predicted *Danaus plexippus* (Dpl) and *Heliconius melpomene* (Hme) butterfly FAD orthologues are labelled in blue and green whereas the desaturase cDNAs characterized from *B. anynana* (Ban-Z11 and Ban-delta9-like) are highlighted in red. Sequence abbreviations correspond to species names as follows: Ape, *Antheraea pernyi*; Ase, *Agrotis segetum*; Ave*, Argyrotaenia velutinana*; Bmo, *Bombyx mori*; Che, *Choristoneura herana*; Cpa, *Choristoneura parrallela*; Dpu, *Dendrolimus punctatus*; Epo, *Epiphyas postvittana;* Has, *Helivocerpa assulta*; Har, *Helicoverpa armigera,* Hvi, *Heliothis virescens;* Hsub, *Helicoverpa subflexa*; Lcap, *Lampronia capitella;* Mbr, *Mamestra brassicae;* Obr, *Operophtera brumata*; Onu, *Ostrinia nubilalis* (Ofu, Ola, Onr.za, Opa, Osc, Oza and Oze correspond to other *Ostrinia* species^45^); Pex*, Planotortrix excessana;* Poc, *Planotortrix octo;* Tni, *Trichoplusia ni*; Tpi, *Thaumetopoea pityocampa*; Yev, *Yponomeuta evonymella* (Yro and Ypa correspond to other *Yponomeuta* species^43^). Numbers at the nodes indicate bootstrap values for 100 replicates.

**Figure 4:**
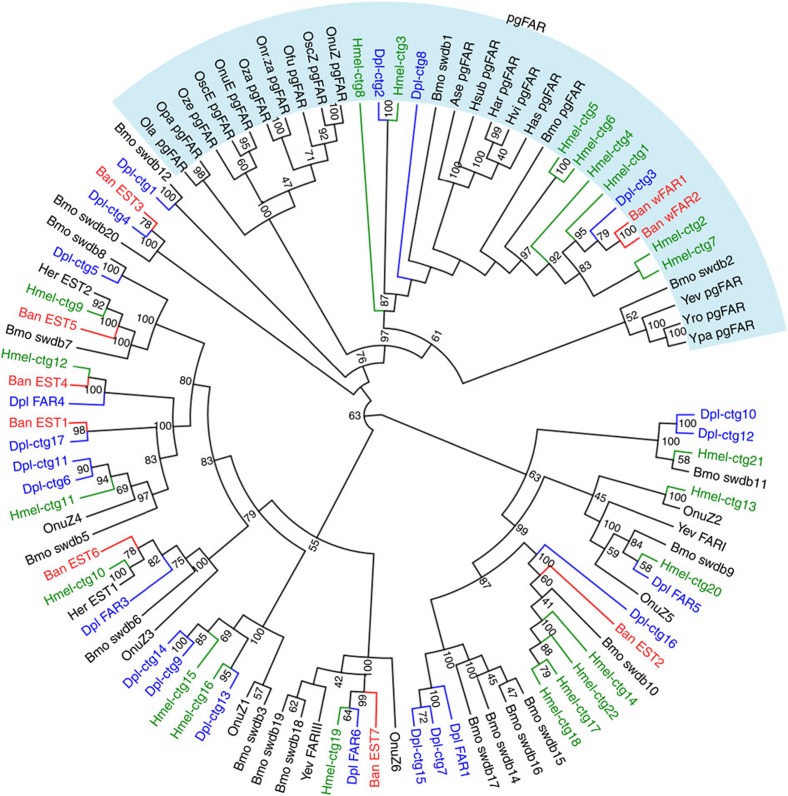
Phylogeny of moths and butterflies FARs. The maximum-likelihood phylogenetic reconstruction was performed using *in silico* predictions for *Danaus* (Dpl) and *Heliconius* (Hmel) as well as the *Bicyclus* FARs, which are highlighted in blue, green and red, respectively, whereas sequences derived from other lepidopteran species are in black. The clade named ‘pgFAR’ represents the subfamily containing FARs functionally active in pheromone biosynthesis. Sequence abbreviations correspond to species names as follows: Ase, *Agrotis segetum*; Bmo, *Bombyx mori*; Has, *Helicoverpa assulta*; Har, *Helicoverpa armigera;* Her, *Heliconius erato*; Hvi, *Heliothis virescens;* Hsub, *Helicoverpa subflexa*; Onu, *Ostrinia nubilalis* (Ofu, Ola, Onr.za, Opa, Osc, Oza and Oze correspond to other *Ostrinia* species^45^); Yev, *Yponomeuta evonymella*; Yro, *Y. rorrella*; Ypa, *Y. padella*. Numbers at the nodes indicate bootstrap values for 100 replicates.

We analysed the available genomes of two Nymphalidae butterflies, the monarch *Danaus plexippus*^51^ and the postman butterfly *Heliconius melpomen*e^9^, which we find to harbour diversified fatty acyl-CoA desaturase (FAD) and FAR gene families (Figs 3 and 4). Both butterflies possess FAD orthologues to all known major dipteran and lepidopteran FAD subfamilies (Fig. 3, Supplementary Fig. 2), including orthologues of the metabolic ancestral Δ9-desaturases (16C>18C) and also of two key pheromone clades so far only identified in moths: the ‘variable’ Δ11-FAD subfamily to which *Hmel*-ctg0, *Hmel*-ctg1 and *Dpl*-ctg1 belong, and the Δ9-FAD (18C>16C) to which belong *Hmel*-ctg2 and *Dpl*-ctg2 (Fig. 3, Supplementary Tables 1 and 2). Nucleic acid and amino-acid sequences corresponding to butterfly *in silico* FAD predictions are provided in Supplementary Data 1.

Whereas *Drosophila melanogaster* harbours seven FAD loci of which *DesatF*, *Desat1* and *Desat2* encode Δ9-desaturases functioning in female and male long-chain cuticular hydrocarbon pheromone synthesis^23^,^24^,^52^, the domesticated silkmoth *Bombyx mori* genome contains at least 16 FAD paralogues (Fig. 3). Of these, four have orthologues in the fruit fly (Bmo-1 is orthologous to *Desat1* and *Desat2*, whereas Bmo-5, Bmo-6 and Bmo-7, respectively, are orthologous to Dmel/CG15531, Dmel/CG9743 and Dmel/CG9747). Of the twelve predicted *B. mori* FAD genes absent in flies, five paralogues derive from an expansion of the Lepidoptera-specific Δ11-like gene subfamily (Fig. 3). With 10 and 11 predicted paralogues, the butterfly desaturase subfamilies thus appear well conserved with the *B. mori* genome (Fig. 3) and each pair of butterfly desaturase orthologues displays a high level of identity at the amino-acid level and a conserved gene structure (Supplementary Fig. 2, Supplementary Table 1). Also, the evolution of the distinct FAD subfamilies in butterflies was likely accompanied by a successive loss of introns (Supplementary Fig. 2), which corroborates earlier investigations of FAD evolution in moths^27^,^31^,^53^,^54^.

Twenty-nine FAR genes were predicted in the Postman and 25 in the Monarch genome, respectively, most of which contain intact open reading frames (Fig. 4, Supplementary Tables 3,4, Supplementary Data 2,3). These findings are in line with the 22 FAR paralogues uncovered in *B. mori* (Fig. 4)^43^,^44^. Interestingly, whereas *B. mori* and *D. plexippus* harbour three gene copies of the moth-specific pheromone production subfamily (*pgFAR*)^44^, that is, the pheromone production enzyme *Bmo*-pgFAR plus two other genes named *Bmo*-swdb1 and *Bmo*-swdb2 for the silkmoth^43^, and *Dpl*-ctg2, *Dpl*-ctg3 and *Dpl*-ctg8 for the Monarch, respectively, *H. melpomene* seems to have undergone a burst of lineage-specific duplications. It harbours eight pgFAR-like orthologues, of which only one gene *Hmel*-ctg8 may be non-functional as a consequence of a frame shift in exon 1. All other predicted pgFAR-like genes (*Hmel*-ctg1 to *Hmel*-ctg7) are contained in two distinct scaffolds presumably as a result from tandem duplications and possess the characteristics of functional FARs.

### Characterization of a male *B. anynana* biosynthetic Δ11-FAD

All FADs including our *in silico* gene predictions of butterfly orthologues share conserved protein motifs (Supplementary Fig. 2). These structural features allowed us to screen the male *Bicyclus* wing transcriptome using oligonucleotide primers targeting histidine-rich regions (HIS1 and HIS3) of desaturase genes in a PCR-based approach^26^. We characterized two full-length cDNAs, one of which shared high sequence conservation with moth Δ9-desaturases (C18>C16) (Fig. 3) and corresponded to a previously identified Δ9-like EST clone (see methods)^55^. In light of our *in vivo* data, this gene is unlikely involved in pheromone biosynthesis. The second cDNA shared high sequence conservation with the predicted Δ11-like genes from *Heliconius* (71 and 67% to *Hmel*-ctg0 and *Hmel*-ctg1, respectively) and *Danaus* (68% to *Dpl*-ctg1) and other previously characterized moth Δ11-desaturases (Fig. 3, Supplementary Fig. 2). Our maximum-likelihood phylogenetic reconstruction (Fig. 3) based on representative members of the different lepidopteran desaturase subfamilies further supports the notion that the *B. anynana* candidate FAD, hereafter named *Ban*-Δ11, is orthologous to members of the pheromone-producing Δ11-desaturase lineage (Fig. 3).

### Characterization of male *B. anynana* pgFAR-like cDNAs

We used the predicted candidate biosynthetic butterfly FAR gene sequences to probe existing EST libraries from *B. anynana*^55^ in search of orthologues of the pheromone FAR family alongside a RACE-PCR cDNA amplification approach followed by Sanger sequencing from male hindwing tissue. We were able to characterize two full-length FAR cDNA candidates, hereafter named *B. anynana* wing FARs 1 and 2 (*Ban*-wFAR1 and *Ban*-wFAR2). These share 76% identity between their coding regions. Both deduced protein sequences display high amino-acid identity with predicted FAR genes from *Danaus* and *Heliconius* (77 and 75% with *Dpl*-ctg3 and 76 and 64% with *Hmel*-ctg1, respectively) and belong to the moth pheromone gland biosynthetic FAR subfamily (pgFARs, Fig. 4).

### Heterologous expression of a butterfly Δ11-FAD ortholog

**Figure 5:**
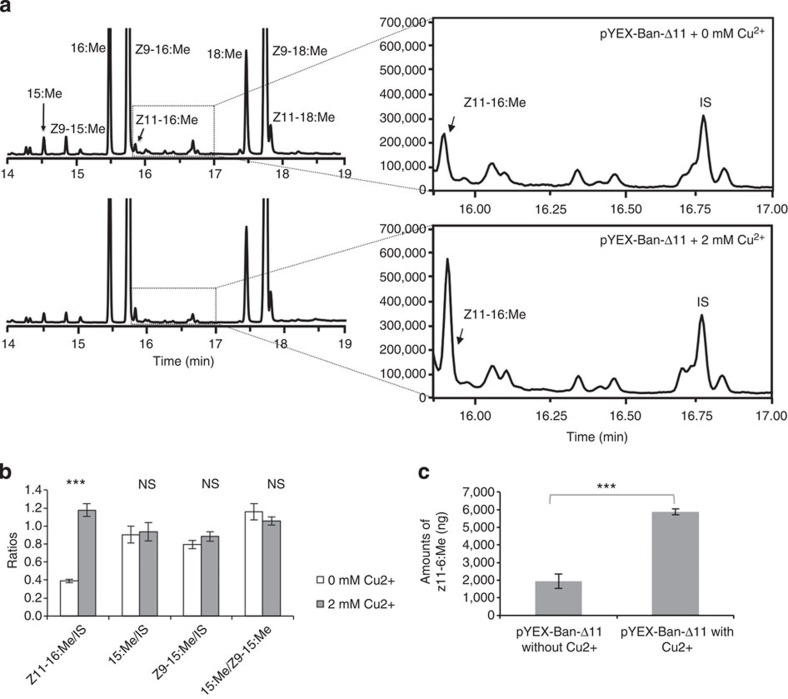
Heterologous expression of *Ban*-Δ11, *B. anynana* pheromone production Δ11-desaturase gene. The graphs in (**a**) represent gas chromatogram traces of FAME from methanolysed *InvSc1 S. cerevisiae* yeast transformed with pYEX-CHT-Ban-Δ11 constructs without Cu_2_SO_4_ (upper panel, 0 mM Cu^2+^-control) and with addition of 2 mM Cu_2_SO_4_ (lower panel). In controls without promoter activation, a minor amount of Z11-16:acid (here and after, in the form of the corresponding methyl ester) is present through chain elongation from Z9-14:acid in yeast, a known activity inherent to the *InvSc1* wild-type yeast strain. (**b**) In pYEX-CHT-Ban-Δ11 Cu^2+^-induced yeast samples, there is a threefold increase in the abundance of Z11-16:acid catalyzed by unsaturation of palmitic acid. Abundance ratios (±s.e.m.) of Z11-16:acid, 15:acid and Z9-15:acid in yeast samples bearing pYEX-*Ban*-Δ11 without (*n*=6) and with Cu^2+^ (*n*=4) relative to the IS (triheptadecenoin; converted into Z10-17:Me during base methanolysis). The *y* axes represent relative abundance. Independent sample *t*-tests were performed in IBM SPSS v.20 to determine whether addition of Cu^2+^ impacted on compound production (Z11-16:acid, ***P<0.001; 15:acid, *P*=0.729 (NS); Z9-15:acid, *P*=0.242 (NS); relative ratio between 15:acid and Z9-15:acid, *P*=0.348 (NS)). (**c**) Total amounts (± s.e.m.) of Z11-16:Me produced by yeast cell cultures bearing pYEX-*Ban*-Δ11 without (*n*=6; 1,948 ng±95) and with Cu^2+^ (*n*=4; 5,881 ng±355) (IBM SPSS v.20, independent samples *t*-test, ***P<0.001).

To test for a biochemical role in male signal production, that is, the ability of *Ban*-Δ11 to catalyse the desaturation of palmitic acid, we placed its ORF under the control of a copper-dependent CUP1 promoter then transformed the resulting pYEX-CHT-*Ban*-Δ11 plasmid in *InvSc1 Saccharomyces cerevisiae* yeast strain and induced protein expression through activation of the CUP1 promoter. Both Δ9-desaturation and elongation occur in the *InvSc1* yeast, leading to the presence of minor amounts of Z11-16:acid (1,948±95 ng) that results from elongation of Z9-14:acid as seen in control yeast extracts (Fig. 5a). After addition of Cu^2+^ however, the relative production of Z11-16:acid consistently increased threefold (5,881±355 ng) in yeast expressing the pYEX-CHT-*Ban*-Δ11 construct (independent samples *t*-test; *P*<0.001) compared with control cultures (Fig. 5b,c). In addition, the relative proportions of endogenous FAs remained constant with or without the addition of copper (15:Me, *P*=0.729; Z9-15:Me, *P*=0.242; relative ratio between 15:Me and Z9-15;Me, *P*=0.348) (Fig. 5b), indicating that *Ban*-Δ11 conferred on the yeast the ability to catalyse the desaturation of palmitic acid and produce higher amounts of Z11-16:acid. No additional monounsaturated compounds were identified, indicating that *Ban*-Δ11 encodes a functional Δ11-desaturase specifically using palmitic acid as a substrate. This result supports the proposed pheromone biochemical pathway towards the production of the intermediate precursor Z11-16:acid.

### Heterologous expression of the butterfly FAR orthologues

**Figure 6:**
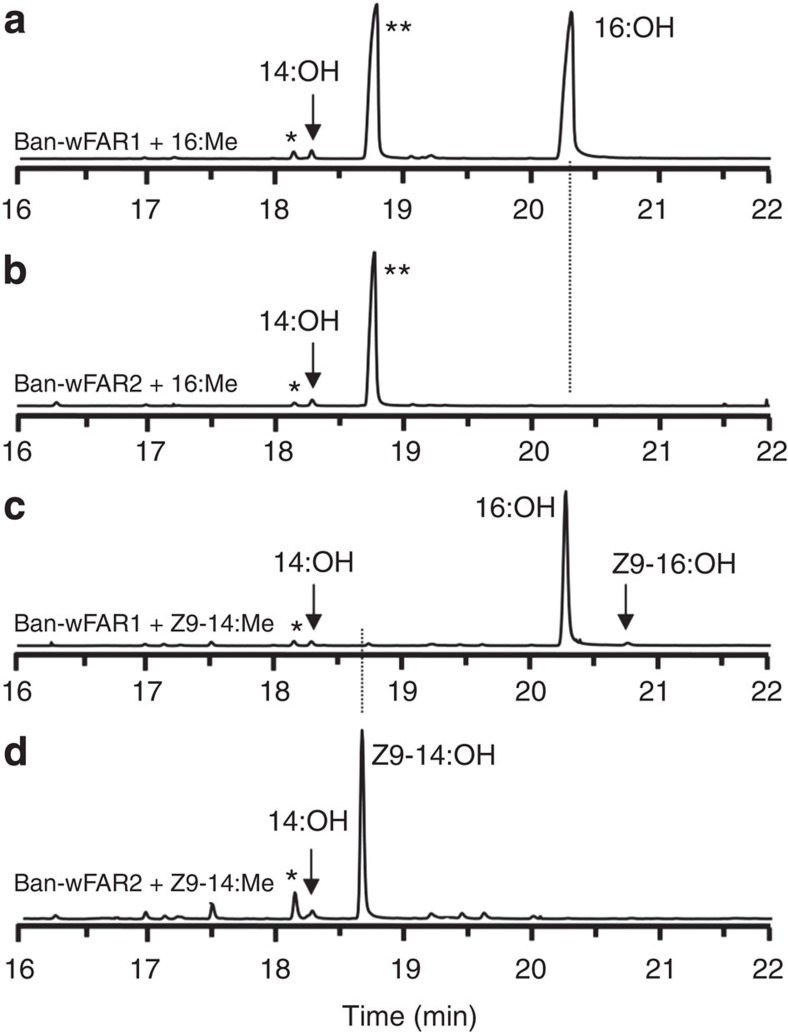
Heterologous expression of *Ban*-wFAR1 and *Ban*-wFAR2, *B. anynana* pheromone production reductase genes. Graphs represent chromatogram traces for total ion currents (TICs) of fatty alcohol extracts from galactose-induced yeast transformed with pYES2.1-*Ban*-wFAR1 and pYES2.1-*Ban*-wFAR2 in presence of 0.5 mM biosynthetic FAME substrates. Presence/absence of alcohols from yeast expressing, from **a** to **d**: (**a**) *Ban*-wFAR1 supplemented with 16:Me, (**b**) *Ban*-wFAR2 supplemented with 16:Me, (**c**) *Ban-*wFAR1 supplemented with Z9-14:Me, (**d**) *Ban*-wFAR2 supplemented with Z9-14:Me. *Ban*-wFAR1 reduces essentially the 16:acid and minor amounts of 14:acid and Z9-16:acid, compounds both naturally present in yeast. Ban-wFAR2 reduces essentially the Z9-14:acid and minor amounts of 14:acid. Asterisks indicate (*) the internal standard (150 ng Z11-13:OH) added alongside hexane extraction, and excess of exogenous 16:acid (**). The *y* axes represent relative abundance.

To determine the putative biological role of the two characterized *B. anynana* biosynthetic FAR cDNA candidates, their respective ORFs were cloned under the control of a GAL1 promoter in the pYES2.1 vector and assayed in *InvSc1* yeast cells. We established earlier that no long-chain fatty alcohols occur naturally in *InvSc1* yeast carrying the control pYES2.1 expression vector, ensuring that the production of alcohol in yeast extracts is conferred by the heterologous insect gene^43^. Yeast cells bearing either gene construct were grown in the presence of galactose and 0.5 mM of individually supplemented Z9-14:Me or 16:Me as biosynthetic precursor. Yeast cells expressing either *Ban*-wFAR1 or *Ban*-wFAR2 catalysed the formation of primary fatty alcohols indicating that both genes are functional FARs (Fig. 6), but with distinct substrate preferences: *Ban*-wFAR1 specifically reduced the 16:Acyl biosynthetic precursor (Fig. 6a,c) towards 16:OH whereas *Ban*-wFAR2 specifically reduced the Z9-14:Acyl towards Z9-14:OH (Fig. 6b,d). *Ban*-wFAR1 produces eight times more 16:OH than *Ban*-wFAR2 produces Z9-14:OH (Supplementary Fig. 3), indicative not only of substrate selectivity but also distinct relative affinity for their respective substrate. *Ban*-wFAR1 is capable of reducing not only exogenous but endogenous 16:acid naturally occurring in yeast (Fig. 6c), and in a fraction of samples, we also detected minor amounts of Z9-16:OH (<2% of the total enzyme alcohol production, Fig. 6c, Supplementary Fig. 3), resulting from conversion of Z9-16:Me naturally occurring in yeast. Finally, both enzymes display a similar minor activity on myristic acid (Fig. 6, Supplementary Fig. 3), which also naturally occurs in yeast. However, in the insect wings myristic acid and Z9-16:acid represent less than 3.5% of the C14-C16 wing lipid content and are not biosynthetic precursors (Figs 1 and 2).

**Figure 7:**
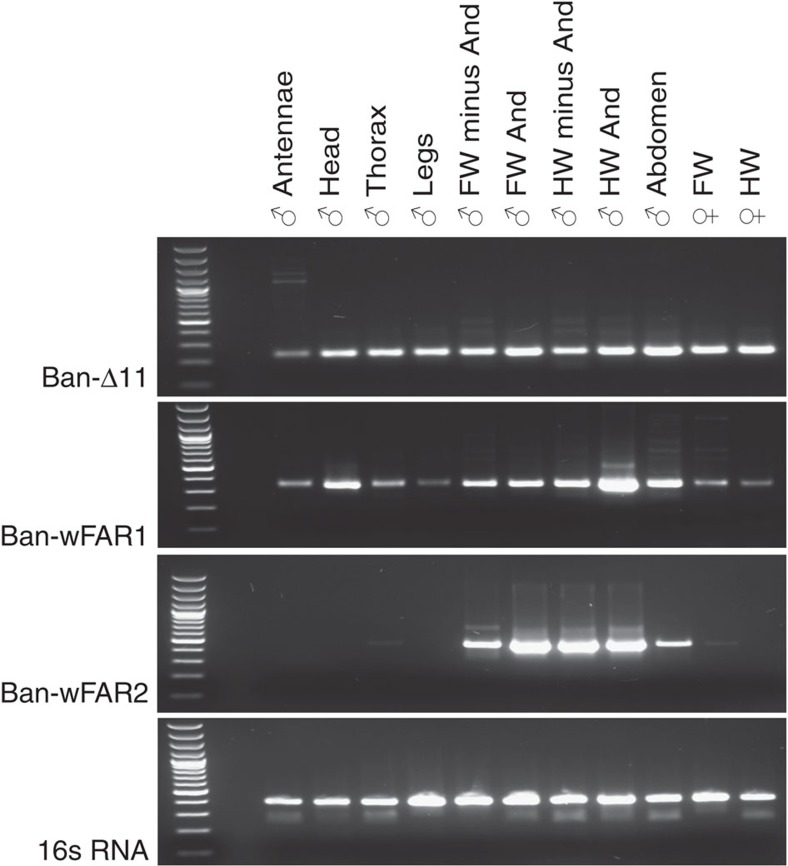
Transcriptional analysis of butterfly wing pheromone genes. Reverse transcription analysis of *B. anynana* Ban-Δ11 and wFAR messenger RNAs. Amplicon sizes: Ban-Δ11, 235 bp; Ban-wFAR1, 336 bp; Ban-wFAR2, 441 bp; 16 s RNA, 397 bp. First wells correspond to a 100-bp DNA ladder (Invitrogen). FW, forewing; HW, hindwing; And, androconia. See also Supplementary Fig. 4.

In addition, semi-quantitative RT–PCR analyses indicated that in contrast to female moths, whose Δ11 and pgFARs implicated in pheromone production typically exhibit pheromone gland-specific expression patterns^43^,^54^, the functional butterfly FAD and FAR transcripts show a broader expression pattern (Fig. 7, Supplementary Fig. 4).

## Discussion

We first investigated the biochemical basis of production of the FA derivatives Z9-14:OH and 16:Ald, the two key pheromone components in *Bicyclus* butterfly males, and demonstrate that they are produced *de novo* along pheromone biosynthetic routes similar to female moths. Our labelling data support that the production of Z9-14:OH in male wings proceeds in a three-step pathway from palmitic acid. Palmitic acid, which is also known as an essential precursor for female moth pheromone biosynthesis^29^, undergoes a desaturation towards Z11-16:acid, followed by one cycle of chain shortening by β-oxidation towards Z9-14:acid and a final reduction to the corresponding alcohol (Fig. 2a). The alternative hypothesis involving a Δ9-desaturase (Fig. 2a) can readily be excluded based on the absence of incorporation from labelled myristic acid. Second, the 16:Ald is synthesized from palmitic acid, via a two-step pathway including reduction to hexadecanol (16:OH), presumably followed by oxidation to the final aldehyde (Fig. 2b). The biochemical pathways identified here clearly suggest that the *Bicyclus* male pheromone components are produced via *de novo* moth-like biosynthetic routes, involving both a FAD and at least one fatty acyl-CoA reductase (FAR).

In order to find genomic evidence of conserved pheromone production pathways and associated biosynthetic genes in butterflies, we mined the available genomes of two Nymphalidae, the monarch and postman butterflies. We show that both of the butterfly genomes harbour diversified FAD and FAR gene families, among which are found orthologues to previously identified lepidopteran pheromone production genes. The butterfly desaturase families appear well conserved with other lepidopteran genomes and in agreement with the evolutionary history of dipteran and lepidopteran ancestors (Fig. 3), and the high diversification of FAR subfamilies in butterflies is concordant with the 22 predicted paralogues in *B. mori* (Fig. 4). There is thus a compelling similarity between the repertoires of pheromone biosynthetic genes of butterflies and moths. This is remarkable considering (i) the loss of long-range female-produced sex pheromones in butterflies, (ii) the chemical unrelatedness of many plant-derived male butterfly pheromonal odours and (iii) the evolutionary timeframe since divergence from a common ancestor.

Functional evidence for shared biosynthetic ancestry of pheromone production between moths and butterflies is first revealed through characterization and heterologous expression of the *B. anynana* butterfly Δ11-desaturase ortholog (Figs 3 and 5). Distinct from cuticular hydrocarbon synthesis in the Diptera^23^, and similar to pheromone biosynthesis in many moth species, we demonstrate that *B. anynana* pheromone production does not proceed through Δ9-desaturation despite the conservation of metabolic^27^ Δ9-FAD gene members in butterflies, and that Δ11-desaturation is of key importance in the genus *Bicyclus*. Δ11-desaturation has previously been recognized as an important innovation of the Lepidoptera that is widely used in sex pheromone biosynthetic pathways in extant ditrysian female moths^27^,^29^,^56^,^57^. This finding thus provides the very first demonstration of the sharing of conserved pheromone-producing genes and biosynthetic modules between female moths and male butterflies across the Lepidoptera.

We next characterize two duplicate genes of the Lepidoptera-specific pgFAR lineage in *B. anynana* (Fig. 4) and demonstrate that they encode specialized pheromone biosynthetic reductases (Fig. 6). The discovery of substrate-specific FAR gene functions may contrast with previously identified broad-range alcohol-producing FARs from the silkmoth^42^, small ermine moths^43^, the majority of corn borer species^44^,^45^ and heliothine moth species^46^, adding to the degree of specialization and range of FAR biosynthetic activities found among lepidopteran species and further demonstrating the conservation and dynamic evolution of ancient biosynthetic networks shared with moths.

Neither the functional *Ban*-Δ11-desaturase nor the *Ban*-wFAR transcripts appear to be male or wing specific (Fig. 7) but their expression is nevertheless consistent with FA precursor distribution as the reaction product of *Ban*-Δ11, the Z11-16:acid and its shortened derivative the Z9-14:acid occur throughout both types of male butterfly wings (Fig. 1). We, however, did not focus specifically on investigating whether these precursors can be found in other adult tissues. In light of the broad messenger RNA distribution pattern of *Ban*-wFAR1, which encodes the enzyme producing the 16:OH, one could speculate that the aldehyde-producing oxidase accounting for the final biosynthetic step is likely to confer the tissue specificity of the aldehyde pheromone component. Finally, *Ban*-wFAR2 encodes a transcript highly expressed in both male forewings and hindwings but not restricted to the androconia (Fig. 7), again in agreement with the presence of Z9-14:OH throughout both types of wings (Fig. 1).

We show that FA desaturases and reductases orthologous to moth-biosynthetic genes are active in a butterfly, specifically *B. anynana*; yet, male abdominal glands of several *Heliconius* butterflies have also been shown to contain a large variety of FA-derived compounds including saturated and unsaturated long-chain alcohol and acetate chemicals^49^,^58^. This suggests that these pathways and enzymes are likely conserved in other butterfly systems. Although no detailed studies have investigated as yet the molecular underpinnings of these secretions, it is realistic to suggest that at least some of the predicted orthologous FAD and FAR genes have remained functional, however different the ethological role that FA derivatives might fulfil in butterflies in general^49^. Altogether, this supports the idea that moth-like biochemical pathways and the underlying genetic networks have survived over long evolutionary times across butterfly lineages to act in the *de novo* biosynthesis of diverse butterfly chemical secretions.

The *Bicyclus* male pheromone has been shown to play a determinant role in female mate choice by guiding their acceptance or rejection of courting males^18^,^19^. Individual males have also been shown to differ in their absolute pheromone titre and ratio at different ages, making the male pheromone composition both a reliable predictor of age and individuality^13^ and a honest signal of an individual male’s characteristics and associated fitness to prospective mates^13^,^18^. Nieberding *et al.*^13^ further demonstrated that the overall increase in the titre of 16:Ald is critical for females to discriminate between young and old males whereas an overall increase in total amounts of Z9-14:OH seems to participate in how females can differentiate young (3 days old) from middle-aged males (14-day to 21 day-old individuals). The two pheromone biosynthetic FARs involved in the pathways towards the production of these two key pheromone components provide an opportunity for uncoupling the male pheromone signals and may contribute to pheromone variation in *Bicyclus*. Age-dependent modulation of male *Bicyclus* signals could take place through changes in FAR enzyme activities and provides the exciting opportunity to further investigate the mechanistic link between male condition, pheromone composition and female choice.

We show that the forewing and hindwing FARs are capable of producing 14:OH as a byproduct of the reduction step and arose following a recent gene duplication event (Fig. 4), suggesting that the ability to reduce 14:Acyl was acquired independently or more parsimoniously that the ancestral copy was less specialized. Hence, FARs characterized from moths so far are often capable of simultaneously reducing C14 and C16 substrates (except in *Ostrinia*^45^), which could imply that the ancestral state in Lepidoptera was in many instances a broad-range FAR that underwent further specialization including this butterfly system. To test whether the ability to produce 16:OH and Z9-14:OH hence follows this subfunctionalization scenario or arose subsequently by neofunctionalization in one or both duplicates will, however, require further comparative molecular and functional analyses. Genetic reshuffling, that is, gene duplication, can provide a mechanism for new pheromones to arise and pheromone variation has been suggested to be positively correlated with the number of genetic elements involved in biosynthetic pathways^59^. Regardless of whether this pattern of specialization at the reduction step is unique to *Bicyclus* or widespread among other butterfly species, gene duplication in this species likely provided the necessary raw material that allowed the dissociation of biosynthetic gene functions. Decreasing functional constraints on gene regulatory elements^60^,^61^ can promote independent variation of gene expression whereas relaxed constraints on the involved gene products could lead to the rapid evolution of new enzymatic functions, respectively, in turn facilitating intraspecific variation in the male signal^62^,^63^.

In conclusion, our central finding, that moth and butterfly lepidopteran lineages share a conserved pheromone production genetic network provides important insights into the evolutionary origin of FA-derived butterfly pheromones, and opens the door to address whether the evolution of dissociated biosynthetic pathways may have contributed to facilitate modulation in the released courtship signals.

## Methods

### Insect rearing

A butterfly population of *B. anynana* (Satyrinae) was established in Lund from hundreds of eggs originating from a lab colony stock originally derived from 80 gravid females collected in Malawi in 1988, and maintained at Leiden and at Yale University since then at a size of about 200–300 breeding individuals each generation.

Larvae were raised in a climate chamber with controlled environmental conditions including a 12L–12D light–dark cycle with 70% relative humidity and 27±1 °C degree Celsius. They fed on regular supplies of fresh young maize plants until pupation. For experiments, newly emerged adults were separated on the day of eclosion (day 0) and held in single-sex cohorts with fresh banana slices as food source.

### Chemicals used for *in vivo* labelling

Deuterium-labelled (16,16,16-D_3_)-hexadecanoic acid (D_3_-16:acid) and (14,14,14-D_3_)-tetradecanoic acid (D_3_-14:acid) were purchased from Larodan Fine Chemicals, Malmö, Sweden. The (*Z*)-(13,13,14,14,15,15,16,16,16-D_9_)-11-hexadecenoic acid (D_9_-*Z*11-16:acid) was available in our chemical database and the (*Z*)-(11,11,12,12,13,13,14,14,14-D_9_)-9-tetradecenoic acid (D_9_-*Z*9-14:acid) was provided by courtesy of Dr J. Millar (UCR, USA).

### Biochemical labelling and biosynthetic pathway

In order to determine the relative wing composition of Z9-14:OH and 16:Ald in the fore- and hindwings, we excised individual forewings or hindwings of 4-day-old individuals and extracted them for 30 min in 1.5 ml glass vials containing n-hexane followed by gas chromatography (GC) analysis as described under the GC–mass spectrometry (GC–MS) analyses section below.

The aldehyde and alcohol biosynthetic pathways were probed using topical wing application of deuterium-labelled FAs. The D_3_-16:acid, D_3_-14:acid, D_9_-*Z*11-16:acid and D_9_-*Z*9-14:acid were dissolved individually in dimethylsulphoxide (DMSO) at a concentration of 20 μg μl^−1^. Natural butterfly movements can cause cross-wing contamination during incubation with the labelled chemicals and therefore, we precisely removed either the fore- or hindwings before topical application on the remaining pair of wings. Insects were anesthetized with carbon dioxide and 1 μl of each single-compound solution was applied to the androconial region, which for forewings constitutes a spot of differentiated scales located on the anal vein on the ventral side^13^.

For hindwing labelling, the labelled compound was applied onto the dorsal side of each hindwing in the region comprised between the subcostal and radial veins^13^. The labelling was carried out 4 h before the onset of scotophase and males were thereafter kept individually for a 24-h incubation period. Labelled androconia from the two hindwings or the two forewings, respectively, were excised and extracted in 200 μl hexane for 30 min. The extracts were analysed by GC–MS using selected ion monitoring (SIM) along with wing extracts from control individuals treated with DMSO only.

To analyse the biosynthetic fatty acyl precursors, wing tissues recovered after the hexane extraction for volatile content analysis were subsequently extracted with 100 μl chloroform:methanol (2:1 v:v) for 24 h at room temperature. The extract was dried under a stream of nitrogen, and the residues were subjected to base methanolysis to convert fatty acyl moieties to the corresponding methyl esters^64^ and subsequently analysed by GC–MS and SIM as described in the next paragraph.

### GC-MS analysis of male wing extracts

Fore- and hindwing hexane extracts and the corresponding FA methyl esters (FAME) were analysed on a Hewlett Packard HP 6890 GC system (Agilent, Palo Alto CA, USA) coupled to a mass selective (MS) detector (HP-5975) equipped with a HP-5MS capillary column (30 m × 0.25 mm, Agilent technologies) with helium as carrier gas and an average velocity of 30 cm s^−1^.

The oven temperature was set at 80 °C, held for 1 min, then increased to 210 °C at a rate of 10 °C min^−1^, held for 12 min and finally increased to 250 °C at a rate of 10 °C min^−1^, held for 5 min. SIM was used to detect the native pheromone components and the corresponding deuterium-labelled compounds. *Z*9-14:OH was detected using the characteristic ions at *m/z* 31, 194 and 212; ions at *m/z* 197 and 203 were used to monitor the corresponding D_3_-*Z*9-14:OH and D_9_-*Z*9-14:OH, respectively. 16:Ald was monitored with the characteristic ions at *m/z* 196 and 222 and the incorporation of D_3_-16:Ald was monitored with ion at *m/z* 199. The identity of all components was confirmed by comparing retention times and mass spectra with those of reference standards.

### Butterfly FAD and FAR gene annotation and phylogeny

The *Danaus* and *Heliconius* butterfly genomes were searched against tBLASTx databases at NCBI^65^ using the deduced protein sequences of active pheromone biosynthetic moth genes as queries. All scaffolds predicted to harbour desaturase and FAR-like genes were retrieved and annotated in Geneious Pro 5.6.4 (ref. 66); intron–exon boundaries were predicted using tBLASTx, Softberry FGENESH and ExPasy prediction tools, manually curated and verified. A number of automated predictions for *Danaus* and *Heliconius* FAD proteins and for *Danaus* FAR proteins were made available through NCBI in the course of this study. For *Danaus* FADs, some automated gene predictions contained verifiable inaccuracies mainly in exon 1, for which we provide the corrected gene structures and associated nt and aa sequences in Supplementary Table 1 and Supplementary Data 1, respectively. For *Danaus* FARs, only a small number of genes were predicted compared with the number retrieved in our extensive *in silico* analysis. Scaffold and structural information for *Heliconius* and *Danaus* FAR genes are listed in Supplementary Tables 3,4, alongside accession numbers corresponding to automated predictions when available, and the associated nt and aa sequences are provided in Supplementary Data 2 and 3, respectively.

Accession numbers for moth FAD and FAR sequences, as well as ESTs, used for the phylogenetic reconstructions are listed in Supplementary Tables 2 and 5 or were given elsewhere^28^,^43^ and are available upon request. For each set of amino-acid sequences, multiple sequence alignments were generated using MAFFT v7 with E-INS-i algorithm and the BLOSUM45 scoring matrix^67^. Maximum-likelihood inference was carried out using the standalone version of PhyML^68^ and the WAG+I+G model as determined by performing model selection in Topali v2.5 (ref. 69)^69^. Clade support was evaluated using 100 bootstrap replicates. The cladograms were visualized and prepared using the online tool EvolView^70^.

### Molecular characterization of FAD and FAR cDNA candidates

First-strand cDNA was synthesized using a Stratascript reverse transcriptase (Stratagene) from 1 μg total RNA extracted from the portion of 6-day-old male hindwings containing the androconia and hair pencils. We opted for screening the transcriptome of this tissue based on the rationale that all candidate biosynthetic genes were to be transcribed in the adult hindwing since it produces both pheromone components.

For the desaturase cDNA screening*, B. anynana* EST libraries^55^ contained only information for a predicted Δ9-candidate desaturase transcript (EST GenBank Acc. nr GE668257 (Ban-delta9-like, Fig. 3). Therefore, we performed a complete PCR-based screen for candidate desaturase genes using male hindwing androconial cDNA as template in PCR reactions with oligonucleotide primers designed against conserved desaturase motifs^31^. PCR thermal cycling conditions consisted of 95 °C for 5 min, 35 cycles at 95 °C for 30 s, 50 °C for 45 s, 72 °C for 90 s and 72 °C for 10 min. An amplicon with expected size of 560-bp was gel purified (Promega), ligated into the pCR2.1-TOPO TA cloning vector system (Invitrogen) and amplified in DH5α *Escherichia coli* cells (New England Biolabs). Purified plasmids were sequenced using the Big Dye Terminator cycle sequencing kit v1.1 followed by analysis on a capillary ABI 3100 sequencer instrument (Applied Biosystems). Double-stranded DNA sequence information encompassing the central fragment of desaturase genes was curated using BioEdit followed by BLAST searches to verify the desaturase gene identity^65^. Clones were identified corresponding to the above-mentioned EST Δ9-desaturase and a Δ11-like desaturase for which 3′-and 5′-cDNA end termini were amplified using the SMART RACE Kit (Clontech) and gene-specific RACE primers (Supplementary Table 6).

For the reductase cDNA screening, BLASTP and TBLASTN searches were used to screen *B. anynana* EST databases using the *Danaus* and *Heliconius* orthologues of biosynthetic FAR candidate genes as queries. Candidate ESTs under Acc. numbers GE680761 and GE680762 (adult head EST library; DOE Joint Genome Institute project ID 16936) were assembled in a partial *B. anynana* contig. The assembled contig clustered with moth-biosynthetic pgFARs and was used as sequence template to design 5′- and 3′-RACE primers in NT1 Vector (Invitrogen) (Supplementary Table 6), which served to obtain the full-length cDNA sequence. The *B. anynana* FAR EST 3′-RACE primer led to the amplification of a 2 kb fragment matching perfectly with the partial EST 5′ region and the corresponding full-length cDNA was named Ban-wFAR1. Twenty-one of the 25 DNA bases from the 3′-RACE primer were conserved enough to amplify a distinct 1 kb long 3′-RACE DNA amplicon, which we found to encode the 3′ cDNA-end of a distinct pgFAR-like transcript as confirmed by phylogenetic analyses. A set of gene-specific 5′-RACE primers was designed to amplify the second clone 5′-cDNA-end, and the compiled full-length cDNA was named Ban-wFAR2. The sequence integrity and distinctness of the two pgFAR-like gene candidates, which share 76% identity at the nt level, was confirmed by amplifying each ORF with gene-specific primers followed by Sanger DNA sequencing.

### Tissue distribution of biosynthetic transcripts

Adult butterfly tissues from fifteen CO_2_-anaesthesized 6-day-old male individuals (head, antenna, thorax, legs, abdomen, forewing androconia, hindwing androconia, fore- and hindwing (minus androconia)) were dissected using microscissors and collected in RNA later. Tissues were also collected from whole fore- and hindwing tissue from fifteen 6-day-old females. Total RNA was isolated using the RNeasy Isolation kit and a DNase purification step (Qiagen). RT–PCR reactions were carried out using the SuperScript III One Step RT–PCR System with Platinum Taq (Invitrogen) in a 25-μl reaction containing 30 ng RNA and 0.8 μM GSP (Supplementary Table 6) and cycling conditions as follows: 55 °C for 30 min, 94 °C for 2 min, 35 cycles of 94 °C for 15 s, 55 °C for 30 s, 68 °C for 45 s and 68 °C for 2 min. PCR products were analysed on a 2% agarose gel. Parallel RT–PCR reactions were Exo-Sap purified and sequenced to confirm gene-specific amplification.

### Functional assay of Ban-Δ11, Ban-wFAR1 and Ban-wFAR2

Gene-specific primers (Supplementary Table 6) were designed to amplify the desaturase and FAR candidate ORFs using male hindwing androconia cDNA as template in combination with the Advantage2 PCR system (Clontech). The desaturase ORF was ligated in the pYEX-CHT expression vector at the BamH1 and EcoR1 restriction sites and each FAR ORF was cloned in the pYES2.1 TA cloning expression vector (Invitrogen) downstream the GAL1 promoter. All constructs were verified by sequencing. The empty pYEX-CHT and pYES2.1 control plasmids as well as the distinct constructs were transformed into the *InvSc1* strain of the yeast *S. cerevisiae* (*MATa his3D1 leu2 trp1-289 ura3-52)* (Invitrogen) and propagated on SC-U plates containing 2% glucose.

For desaturase expression in pYEX-CHT, yeast prototrophs were selected and grown in 20 ml SC-U 2% glucose and constant agitation for 48 h at 30 °C and 300 r.p.m. in a shaking incubator prior to dilution to an OD_600_ of 0.4 in 250 ml flasks containing 10 ml fresh selective medium. At this stage, yeast cultures of single transformants were run in parallel with or without addition of 2 mM Cu^2+^ (resulting in CUP promoter induction), that is, the addition of 20 μl CuSO_4_ 1 M. Equal amounts of cells (as measured by their OD) from CUP-induced and CUP-non-induced yeast cultures were harvested by centrifugation and the supernatant was discarded. The total lipid fraction in the yeast pellet was extracted with 0.5 ml chloroform:methanol (2:1; v-v) spiked with 10 ng ul^−1^ triheptadecenoin (Larodan, Sweden) as internal standard, to extract the total cell lipid content prior to base methanolysis^28^. Hexane samples were stored at −20 °C until GC-MS analyses (See GC–MS section described below).

The FAR functional assay was performed following a protocol explained in detail elsewhere^43^ with alcohol-free Z9-14:Me and 16:Me (Larodan) used as biosynthetic substrates in the yeast assays. Briefly, individual prototroph colonies were inoculated in 5 ml SC-U medium and incubated for 48 h at 30 °C and 300 r.p.m. (Innova 42, New Brunswick Scientific), diluted to an OD600=0.4 to a final volume of 20 ml SC-U 2% galactose and 0.1% glucose in 250-ml flasks, and incubated for 24 h at 30 °C and 200 r.p.m. Yeast cultures were diluted to 1:10 in 2 ml SC-U 2% galactose, 1% tergitol (Nonidet P-40, Sigma), and 0.5 mM alcohol-free precursors in the form of methyl esters. After incubation for 24 h at 30 °C and 300 r.p.m., cells were collected by centrifugation at 2,000 r.p.m. (Labofuge 200, Heraeus Instruments) and washed in sterile water. Cell pellets were extracted with 1 ml *n*-hexane spiked with 150 ng Z11-13:OH as an internal standard followed by shaking at 200 cycles/min (Vibramax 100, Heidolph) for 60 min. Hexane samples were stored at −20 °C until GC-MS analysis.

### GC–MS analysis of yeast extracts

Prior to analysis, hexane extracts from base-methanolysed *InvSc1* yeast cultures expressing the pYEX control, and the pYEX-Ban-Δ11 constructs with or without Cu^2+^ induction were concentrated under a gentle flow of pure nitrogen to a final volume of 25 μl and transferred into 1.5-ml vials containing glass inserts. One microlitre was injected on a Hewlett Packard HP 6890 GC system coupled to an automatic injector (HP-7683) and a mass selective (MS) detector (HP-5975). The GC was equipped with a polar HP-INNOWax column (100% polyethylene glycol, 30 m × 0.25 mm × 0.25 μm; Agilent Technologies) with helium as carrier gas and an average velocity of 30 cm sec^−1^. The MS was operated in electron impact mode (70 eV), the GC oven temperature was set at 50 °C for 2 min and then rose at a rate of 10 °C min^−1^ up to 220 °C, held for 20 min.

For analysis of hexane extracts from *InvSc1* yeast cultures expressing the pYES-only, the pYES-BanFAR1 or pYES-BanFAR2 construct, extracts were concentrated to 50 μl. Two microlitres were manually injected on a gas chromatograph (Hewlett Packard HP 5890II GC system) coupled to a mass selective detector (HP 5972) and equipped with a polar INNOWax column (100% polyethylene glycol, 30 m × 0.25 mm × 0.25 μm, Agilent Technologies). The GC–MS was operated in electron impact mode (70 eV) and the GC injector was configured in splitless mode at 220 °C with helium used as carrier gas (average velocity: 20 cm s^−1^). The oven temperature was set at 50 °C for 2 min and then rose at a rate of 10 °C min^−1^ up to 220 °C, with a final hold at 220 °C for 20 min.

## Author contributions

M.A.L., J.-M.L., and C.L. designed the research. H.-L.W. and J.-M.L. conceived and carried out the biochemical experiment and H.-L.W. analysed the data. M.A.L. conceived and carried out the molecular and functional experiments and analysed the data. M.A.L. and J.-M.L. performed the bioinformatics and phylogenetic analyses. M.A.L. wrote the manuscript with contributions from all authors. All authors discussed the results and approved the final version.

## Additional information

**Accession Codes**: The *B. anynana* FAD and FAR sequences reported in this paper have been deposited in the GenBank nucleotide database under accession codes JQ978770 to JQ978773.

**How to cite this article**: Liénard, M. A. *et al.* Sex pheromone biosynthetic pathways are conserved between moths and the butterfly *Bicyclus anynana*. *Nat. Commun.* 5:3957 doi: 10.1038/ncomms4957 (2014).

## Supplementary Material

Supplementary Information Supplementary Figures 1-4, Supplementary Tables 1-6 and Supplementary References

Supplementary Data 1 Nucleic acid and amino acid sequences corresponding to *Danaus plexippus and Heliconius melpomene* FAD contigs.

Supplementary Data 2 Nucleic acid and amino acid sequences corresponding to *Heliconius melpomene* FAR contigs

Supplementary Data 3 Nucleic acid and amino acid sequences corresponding to *Danaus plexippus* FAR contigs.
