# Supplementary material for: Sex pheromone biosynthetic pathways are conserved between moths and the butterfly Bicyclus anynana
Source: Nat Commun. 2014 May 27;5:3957. doi: 10.1038/ncomms4957 (PMC4050330; doi:10.1038/ncomms4957)
Supplement: Supplementary Information — Supplementary Figures 1-4, Supplementary Tables 1-6 and Supplementary References [file ncomms4957-s1.pdf]

Supplementary Figure 1

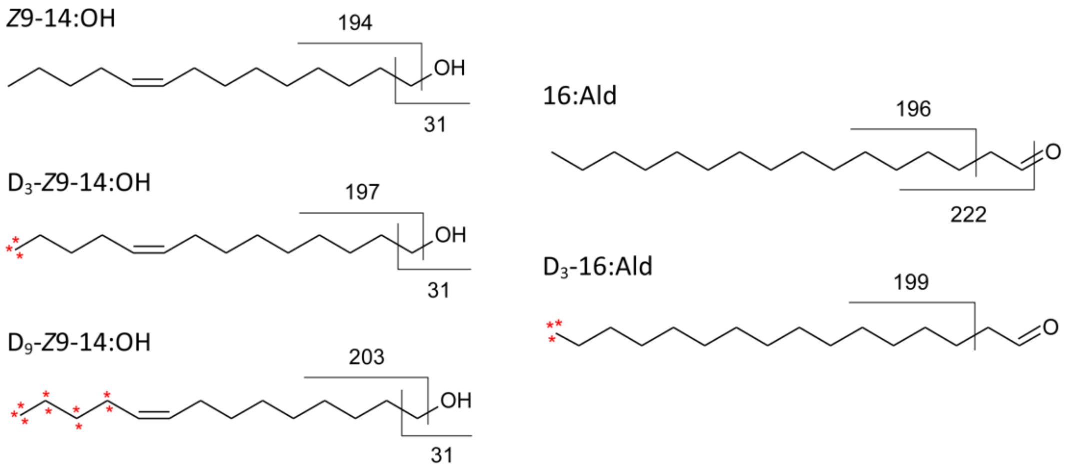

Structures of native and labelled chemical FA compounds used for *in vivo* wing labelling. The structural schemes indicate the proposed fragmentation and diagnostic ions used for monitoring *in vivo* incorporation. Deuterium labels are represented as asterisks.

Supplementary Figure 2

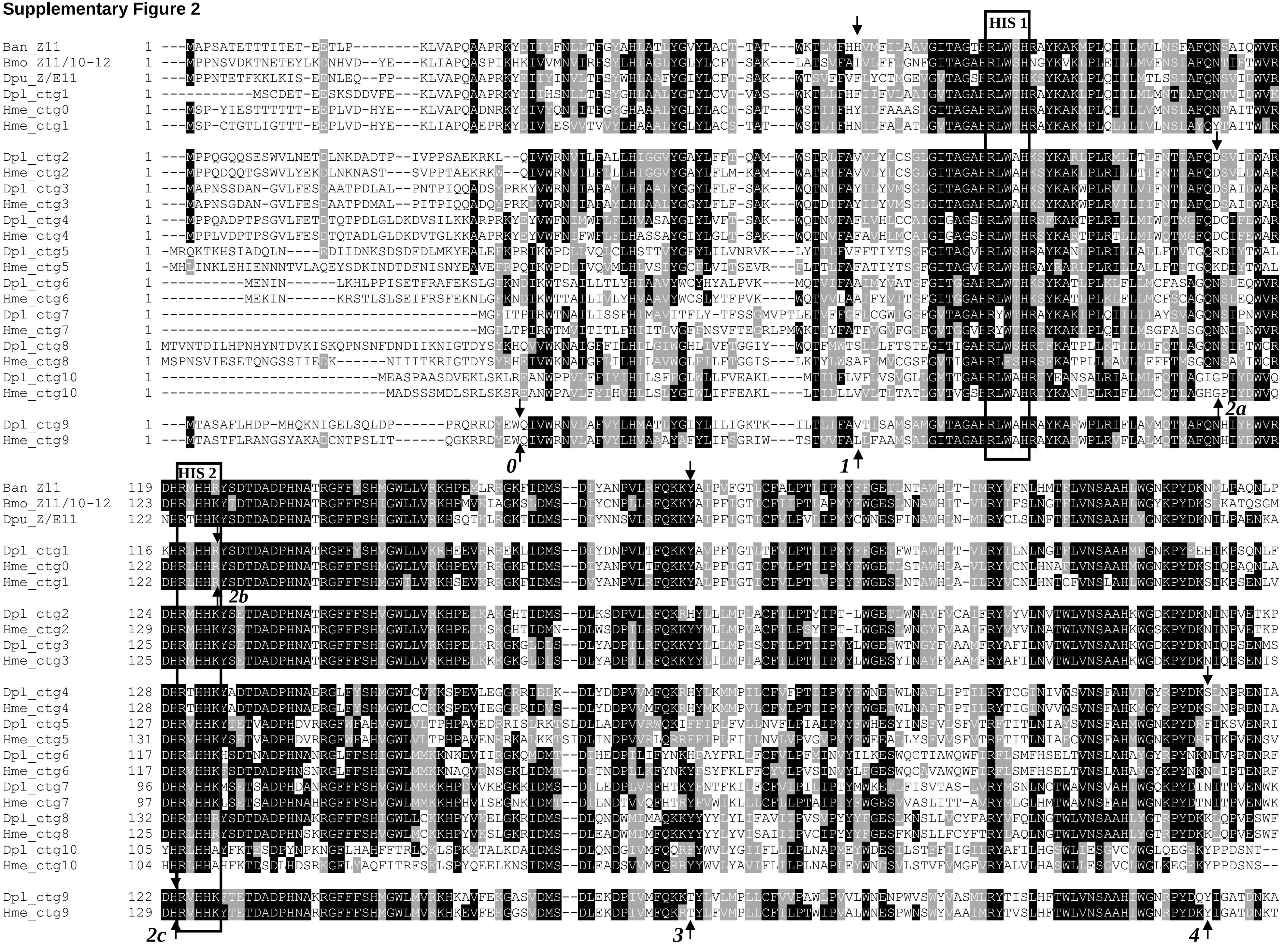

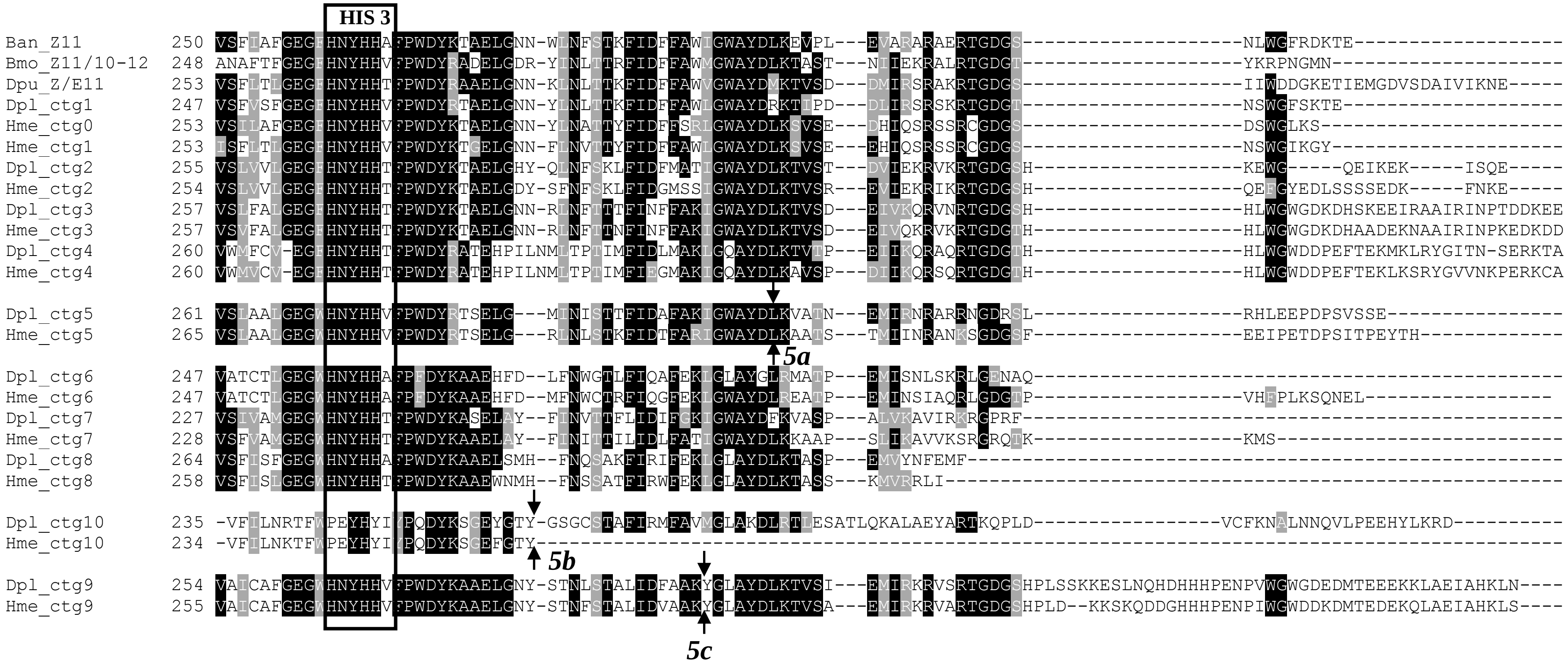

Amino-acid alignment and intron positions of fatty-acyl-CoA desaturases from moth and butterflies. Black, grey and white backgrounds indicate amino acid conservation, semi- or non-conservative substitutions (Boxshade 3.21, ExPASy). Intron positions are located at upper and lower black arrows. All sequences delineated by two arrows share the corresponding intron position, whereas others lack the specific intron. Abbreviated species names correspond to: Ban, *Bicyclus anynana*; Bmo, *Bombyx mori* (Acc. nr. BAD18122); Dpl, *Danaus plexippus*; Dpu, *Dendrolimus punctatus* (Acc. nr. EU152400) and Hme, *Heliconius melpomene*. Information corresponding to the predicted FAD contigs including scaffold numbers and accession numbers are listed in Supplementary Tables 1 and 2, and the corresponding nt/aa sequences are available in Supplementary Data 1. Boxed regions refer to Histidine motifs (HIS 1, HIS2 and HIS3). The Dpu-Z11 and Bmo-Z11/10-12-desaturases possess the two-intron pattern (*1* and *3*) typical of the Δ11-desaturase subfamily. Butterfly desaturase orthologs to Ban-Z11 (ctg0 and ctg1) have an additional intron (*2b*) located in the HIS2 domain. Dpl and Hme orthologs to the two classical Δ9 desaturase C16 > C18 and C18 > C16 subfamilies (ctg2 and ctg3) display a typical three-intron pattern (*1, 2a, 3*) whereas all other putative desaturase genes (ctg4 to ctg10) possess a fourth intron (*4*) prior HIS3, which is conserved in both *Drosophila melanogaster* desat1 and desat2 genes [1,2]. Additional intron *2c* is found in ctg9 and introns *5a, 5b* or *5c* are found in ctg5, ctg10 and ctg9, respectively. Small case letters

indicate introns with variable positions in distinct subfamilies, whose positions do not differ by more than 15-aa (e.g. intron 2a,b and c or intron 5a,b,c). All but Hme_ctg10 deduced aa sequences are complete.

Supplementary Figure 3

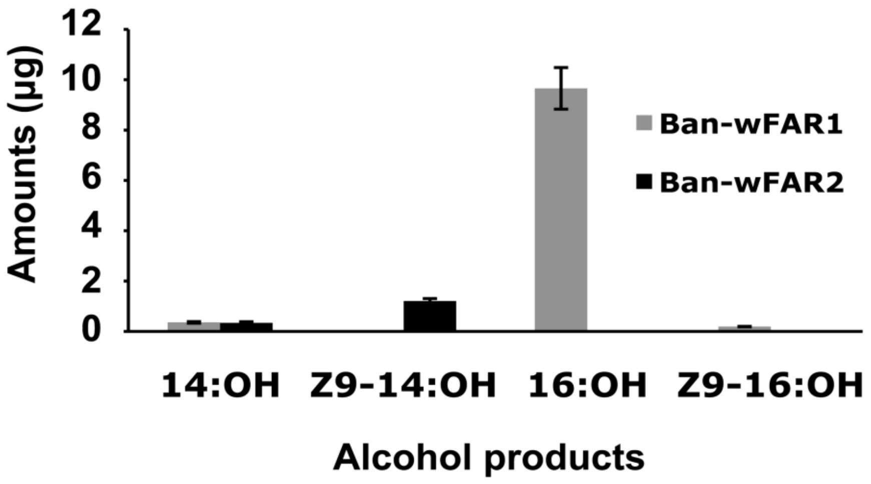

Absolute amounts of fatty alcohols produced by *Bicyclus anynana* wing reductases Ban-wFAR1 and Ban-wFAR2. The two FAR proteins expressed in the Invsc1-pYES yeast expression system exhibit distinct substrate preferences. Ban-wFAR1 preferentially reduces palmitic acid (16:Me) into palmityl alcohol (16:OH; average production: 9657 ng ± 827) compared to the conversion of myristic acid (14:Me) into myristoleyl alcohol (14:OH; 358 ng ± 31) and (*Z*)-9-hexadecenoic acid into palmitoleyl alcohol (Z9-16:OH; 193 ng ± 9). Ban-wFAR2 preferentially produces (*Z*)-9-tetradecenol (Z9-14:OH; 1207 ng ± 98) compared to 14:OH (341 ng ± 44). All alcohol amounts are expressed relative to the Z11-13:OH (internal standard). Bars represent the s.e.m for four independent yeast cultures.

Supplementary Figure 4

A) Ban-wFAR1 and wFAR2 B) 16S RNA and Ban-delta9-like C) Ban-Δ11

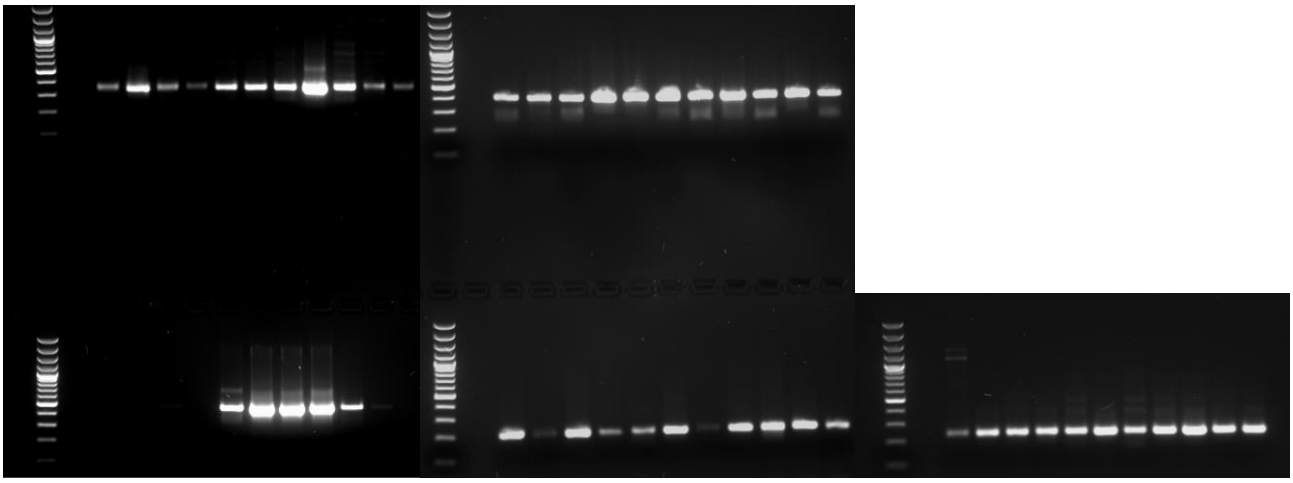

Whole agarose gel pictures associated with Fig. 7 showing the reverse transcription analysis of *B. anynana*. (A) RT-PCR analysis of Ban-wFAR1 (upper part of the gel) and Ban-wFAR2 (lower part of the gel), (B) RT-PCR analysis of 16S RNA (upper part of the gel) and Ban-delta9-like (lower part of the gel, not included in Fig 7), (C) RT-PCR analysis of Ban-Δ11. First wells correspond to the 100-bp DNA ladder (Invitrogen) displayed from 100 bp (lower band) to 1,500 bp (higher band). Second wells are empty. Adjacent wells in each panel from left to right represent the following tissues (as shown in Fig. 7): male antennae, male head, male thorax, male legs, male forewing minus androconia, male forewing androconia, male hindwing minus androconia, male hindwing androconia, male abdomen, whole female forewing, whole female hindwing. Amplicon sizes: Ban-Δ11, 235 bp; Ban-wFAR1, 336 bp; Ban-wFAR2, 441 bp; 16s RNA, 397 bp. The RT-PCR was performed simultaneously for all target genes and tissues, and the whole reaction volume for each sample was loaded on 2% agarose gels.

Supplementary Table 1

Whole genome shotgun sequence accession number, position, length and name attributed to curated butterfly gene contigs used to reconstruct the desaturase phylogeny

Species name	Sequence name[a]	Scaffold number	Position (orientation)	Deduced protein length (aa)	Number of Exons	Position of exons (exon #)
Danaus plexippus	Dpl-KPSQ-ctg1	AGBW01001852	22,822-26,661 (+)	325	4	22,822-23,011(1); 23,551-23,725(2); 24,872-25,033(3); 26,214-26,661(4)
	Dpl-NPVE-ctg2	AGBW01013917[b]	131,056-134,890 (+)	339	4	131,056-131,269(1); 131,814-131,944(2); 133,638-133,843(3); 134,425-134,890(4)
	Dpl-QPSE-ctg3	AGBW01002660	84,418-79,259 (-)	356	4	84,418-84,202(1); 84,040-83,910(2); 80,278-80,073(3); 79,772-79,259(4)
	Dpl-NPRE-ctg4	AGBW01007311[b]	2,847-965 (-)	358	6	2,847-2,622(1); 2,548-2,418(2); 2,326-2,121(3); 2,044-1,859(4); 1,708-1,552(5); 1,132-965(6)
	Dpl-KSVE-ctg5	AGBW01005953	8,462-18,467(+)	342	6	8,462-8,684(1); 9,470-9,600(2); 17,195-17,406(3); 17,671-17,856(4); 17,965-18,134(5); 18,364-18,467(6)
	Dpl-VPRE-ctg6	AGBW01004204	180,755-178,472 (-)	316	5	180,755-180,569(1); 180,445-180,315(2); 179,833-179,628(3); 179,120-178,935(4); 178,709-178,472(5)
	Dpl-TPVE-ctg7	AGBW01009502	69,117-65,274 (-)	295	5	69,117-68,988(1); 67,577-67,447(2); 66,470-66,265(3); 66,034-65,852(4); 65,507-65,274(5)
	Dpl-QPVE-ctg8	AGBW01005024[b]	92,315-93,635 (+)	327	5	92,315-92,552(1); 92,664-92,794(2); 92,867-93,072(3); 93,141-93,326(4); 93,416-93,635(5)
Heliconius melpomene	Hme-QPAQ-ctg0	CAEZ01004008	61,766-65,783 (+)	329	4	61,766-61,973(1); 63,176-63,350(2); 63,607-63,768(3); 65,342-65,783(4)
	Hme-KPSE-ctg1	CAEZ01001764	18,811-16,997 (-)	330	4	18,811-18,604(1); 18,260-18,086(2); 17,836-17,675(3); 17,441-16,997(4)
	Hme-NPVE-ctg2	CAEZ01010151	1,859-5,906 (+)	343	4	1,859-2,069(1); 2,551-2,681(2); 4,819-5,024(3); 5,426-5,906(4)
	Hme-QPSE-ctg3	CAEZ01008204	30,527-27,565 (-)	356	4	30,527-30,311(1); 29,408-29,278(2); 28,557-28,352(3); 28,078-27,565(4)
	Hme-NPRE-ctg4	CAEZ01011218	41,024-38,730 (-)	359	6	41,024-40,799(1); 40,712-40,582(2); 40,212-40,007(3); 39,648-39,463(4); 39,186-39,030(5); 38,900-38,730(6)
	Hme-PVEN-ctg5	CAEZ01010146	16,166-11,283 (-)	349	6	16,166-15,931(1); 15,424-15,294(2); 13,376-13,165(3); 12,494-12,309(4); 12,197-12,028(5); 11,395-11,283(6)
	Hme-PTEN-ctg6	CAEZ01006121	208,007-205,653 (-)	333	5	208,007-207,803(1); 207,383-207,253(2); 206,971-206,766(3); 206,442-206,257(4); 205,923-205,653(5)
	Hme-TPVE-ctg7	CAEZ01010146	7,163-9,758 (+)	300	5	7,163-7,295(1); 7,656-7,786(2); 8,357-8,562(3); 9,087-9,269(4); 9,512-9,758(5)
	Hme-QPVE-ctg8	CAEZ01000036	19,868-22,358 (+)	319	5	19,868-20,087(1); 20,574-20,704(2); 21,280-21,485(3); 21,755-21,940(4); 22,145-22,358(5)

[a] Protein accession numbers made available through NCBI during the course of this study that match our manually curated Dpl-ctg 3 (EHJ76461), Dpl-ctg4 (EHJ68613), Dpl-ctg5 (EHJ70677), Dpl-ctg6 (EHJ73552) and Dpl-ctg7 (EHJ66504). Other conceptual predictions from NCBI with accession numbers EHJ77791, EHJ63142 and EHJ69993 had inaccuracies in their 5'or 3' ends and are corrected as Dpl-ctg1, ctg2 and ctg8. [b] Alternative scaffolds containing partial of full sequence information for Dpl-NPVE: AGBW01008476, for Dpl-QPVE:AGBW01006456 and for Dpl-NPRE: AGBW01007536 (230-2,118 (+)).

Supplementary Table 2

List of species names and corresponding published accession numbers for sequences used in the FAD phylogeny

Gene abbreviation	Species Name	Accession number
Ape_Z/E6	*Antheraea pernyi*	GU952764
Ape_Z11	*Antheraea pernyi*	GU952763
Ave_Z/E11	*Argyrotaenia velutinana*	AAL16642
Ave_Z9	*Argyrotaenia velutinana*	AAF44709
Ban-delta9-like	*Bicyclus anynana*	JQ978773
Bmo_Z11/10,12	*Bombyx mori*	BAD18122
Bmo-1	*Bombyx mori*	AFK13829
Bmo-2	*Bombyx mori*	AF182406
Bmo-3	*Bombyx mori*	AGO45851
Bmo-4	*Bombyx mori*	AGO45856
Bmo-5	*Bombyx mori*	AGO45855
Bmo-6	*Bombyx mori*	AGO45854
Bmo-7	*Bombyx mori*	BGIBMGA010614PA
Bmo-8	*Bombyx mori*	AGO45853
Bmo-9	*Bombyx mori*	BAD18124
Bmo-10	*Bombyx mori*	AGO45852
Bmo-11	*Bombyx mori*	ABD36148
Bmo-12	*Bombyx mori*	AGO45857
Bmo-13	*Bombyx mori*	AGO45858
Bmo-14	*Bombyx mori*	AGO45859
Bmo-15	*Bombyx mori*	XP_004933819
Che_Z/E6	*Choristoneura herana*	JN022486
Cpa_E11	*Choristoneura parallela*	AAQ12891
Cpa_Z9	*Choristoneura parallela*	AAQ12887
Cro_Z9	*Choristoneura rosaceana*	AAN39697
Dpl_ctg9	*Danaus plexippus*	EHJ71380
Dpl_ctg10	*Danaus plexippus*	EHJ66506*
Dpu	*Dendrolimus punctatus*	EU152405
Dpu_Z/E11(1)	*Dendrolimus punctatus*	EU152399
Dpu_Z/E11(2)	*Dendrolimus punctatus*	EU152400
Dpu_Z/E9	*Dendrolimus punctatus*	EU152401
Epo_E11	*Epiphyas postvittana*	AAL11496
Epo_Z9	*Epiphyas postvittana*	AAL35750
Epo_Z9_b	*Epiphyas postvittana*	AAK94070
Has	*Helicoverpa assulta*	AAM28480
Has_Z11	*Helicoverpa assulta*	AAM28483
Has_Z9	*Helicoverpa assulta*	AAM28481
Has_Z9_b	*Helicoverpa assulta*	AAM28484
Hme_ctg9	*Heliconius melpomene*	HMEL015726-PA
Hme_ctg10	*Heliconius melpomene*	HMEL002311-PA**
Lca_NF	*Lampronia capitella*	EU152334
Lca_Z11	*Lampronia capitella*	EU153335
Lca_Z9	*Lampronia capitella*	EU152332
Mbr_Z11	*Mamestra brassicae*	ABX90049
Mse_Z11/10,12	*Mamestra brassicae*	CAJ27976
Mse_Z9	*Manduca sexta*	CAJ27975
Obr_tdes	*Operophtera_brumata*	H0917684
Ofu_Z/E14	*Ostrinia furnacalis*	AAL35746
Onu_Z/E11	*Ostrinia nubilalis*	AAL35331
Onu_Z/E14	*Ostrinia nubilalis*	AAL35330

Onu_Z9	*Ostrinia nubilalis*	AAF44710
Onu_Z9_b	*Ostrinia nubilalis*	AAL29454
Pex_tdes	*Planotortrix excessana*	JN022483
Poc_Z10	*Planotortrix octo*	AAG54077
Poc_Z9	*Planotortrix octo*	AAF73073
Sli_Z11/10,12	*Spodoptera littoralis*	AAQ74259
Sli_Z9	*Spodoptera littoralis*	AAQ74258
Sli_Z9_b	*Spodoptera littoralis*	AAQ74257
Tni_Z11	*Trichoplusia ni*	AF035375
Tni_Z9	*Trichoplusia ni*	AAB92583
Tpi_Z11/11,13	*Thaumetopoea pityocampapi*	ABO43722
Yev_Z/E11	*Yponomeuta evonymella*	HM636633
Ypa_Z/E11	*Yponomeuta padella*	HM636634

* Manual correction for the predicted Dpl-ctg10. Exon 1 from Scaffold AGBW01009502 corrected to AGBW01009502.1:79435…79951

** 3'end partial

Supplementary Table 3 (1/4)

Whole genome shotgun sequence accession numbers, position, length and name attributed to *H. melpomene* gene contigs (Hmel) used to reconstruct the FAR phylogeny

Sequence name	Scaffold number (DBsource)	Length (Complete/partial)	Scaffold Position (orientation)	Deduced aa length	Total number of Exons[3]
Hmel-ctg1	CAEZ01002810	Complete	69,211-75,668 (+)	444	10
Hmel-ctg2	CAEZ01002810	Complete	78,759-87,404 (+)	446	10
Hmel-ctg3	CAEZ01002810	Complete	94,168-88,088 (-)	457	10
Hmel-ctg4	CAEZ01002811	Complete	15,357-6,961 (-)	463	10
Hmel-ctg5	CAEZ01002811	Complete	59,136-53,913 (-)	458	10
Hmel-ctg6	CAEZ01002811	Complete	76,909-71,118 (-)	458	10
Hmel-ctg7	CAEZ01002811	Complete	26,581-19,317 (-)	446	10
Hmel-ctg8[1]	CAEZ01007863	Complete	52,912-44,988 (-)	478	10
Hmel-ctg9	CAEZ01008291	Complete	41,498-37,599 (-)	466	10
Hmel-ctg10	CAFA01012750	Complete	6,939-14,330 (+)	481	12
Hmel-ctg11	CAEZ01009099	Complete	19,029-13,891 (-)	441	10
Hmel-ctg12	CAEZ01009099	Complete	6,505-1,487 (-)	464	11
Hmel-ctg13	CAEZ01009733	Complete	35,582-26,646 (-)	503	11
Hmel-ctg14	CAEZ01009731	Complete	22,435-9,365 (-)	520	11
Hmel-ctg15	CAEZ01004901	Complete	4,680-11,766 (+)	518	13
Hmel-ctg16	CAEZ01004900	Complete	1,347-7,901(+)	423	12
Hmel-ctg17	CAEZ01009104	Complete	22,691-15,590 (-)	524	10
Hmel-ctg18	CAEZ01009104	Complete	8,692-1,437 (-)	479	11
Hmel-ctg19	CAEZ01009816	Partial	168-5,353 (+)	407 [2]	nd

Supplementary Table 3 (2/4)

Sequence name	Scaffold number (DBsource)	Full-length	Scaffold Position (orientation)	Deduced aa length	Total number of Exons[3]
Hmel-ctg20	CAEZ01004442	Partial	295-4,825 (+)	409 [2]	nd
Hmel-ctg21	CAEZ01006908	Complete	6,308-1,014 (-)	466	10
Hmel-ctg22[1]	CAFA01005670	Partial	11,387-4,083 (-)	483 [2]	nd
Hmel-ctg23	CAEZ01010758	Partial	15,636-10,119 (-)	317 [2]	nd
Hmel-ctg24	CAEZ01002136	Partial	625-4,810 (+)	305 [2]	nd
Hmel-ctg25	CAEZ01009101	Partial	1,060-5,482 (+)	228 [2]	nd
Hmel-ctg26	CAEZ01009815	Partial	7,942-11,375 (+)	202 [2]	nd
Hmel-ctg27	CAEZ01009100	Partial	14,107-15,238 (+)	132 [2]	nd
Hmel-ctg28	CAFA01006575	Partial	3-1,962 (+)	160 [2]	nd
Hmel-ctg29	CAFA01002705	Partial	295-2,496 (+)	194 [2]	nd

[1]Pseudogenes due to missense mutations in predicted coding exons, or due to missing internal exons or partial genes that do not fall over scaffold boundaries, [2] Partial deduced aa sequences, [3] Only assigned for full-length FAR-like proteins; nd = not determined

Supplementary Table 3 (3/4)

Sequence name	Position of exons (exon #)
Hmel-ctg1	69,211-69,285(1); 69,952-70,068(2); 70,374-70,496(3); 71,587-71,712(4); 72,133-72,277(5); 72,710-72,811(6); 73,422-73,608(7); 73,906-74,012(8); 74,841-74,970(9); 75,449-75,668(10)
Hmel-ctg2	78,759-78,839(1); 80,688-80,804(2); 81,179-81,301(3); 81,802-81,927(4); 82,278-82,422(5); 82,649-82,747(6); 83,392-83,578(7); 83,814-83,920(8); 84,705-84,834(9); 87,182-87,404(10)
Hmel-ctg3	94,168-94,067(1); 92,130-92,014(2); 91,923-91,801(3); 91,421-91,296(4); 91,130-90,977(5); 90,694-90,593(6); 90,074-89,888(7); 89,375-89,269(8); 89,075-88,946(9); 88,310-88,088(10)
Hmel-ctg4	15,357-15,238(1); 14,570-14,454(2); 13,872-13,750(3); 13,288-13,163(4); 10,881-10,737(5); 10,261-10,160(6); 9,861-9,675(7); 9,330-9,218(8); 8,793-8,664(9); 7,186-6,961(10)
Hmel-ctg5	59,136-59,017(1); 58,267-58,151(2); 57,280-57,158(3); 56,889-56,764(4); 56,506-56,393(5); 55,731-55,630(6); 55,409-55,223(7); 54,999-54,893(8); 54,393-54,289(9); 54,132-53,913(10)
Hmel-ctg6	76,909-76,790(1); 75,110-74,994(2); 74,633-74,511(3); 74,183-74,144(4); 73,721-73,577(5); 73,246-73,145(6); 72,909-72,723(7); 72,499-72,393(8); 71,573-71,447(9); 71,337-71,118(10)
Hmel-ctg7	26,581-26,501(1); 26,188-26,072(2); 25,679-25,557(3); 24,891-24,766(4); 22,772-22,628(5); 21,613-21,556(6); 21,133-20,947(7); 20,361-20,255(8); 19,982-19,853(9); 19,536-19,317(10)
Hmel-ctg8 [1]	52,912-52,739(1); 52,418-52,305(2); 51,661-51,539(3); 48,521-48,433(4); 48,096-47,949(5); 47,554-47,453(6); 46,906-46,720(7); 46,260-46,154(8); 45,887-45,758(9); 45,210-44,988(10)
Hmel-ctg9	41,498-41,424(1); 41,423-41,307(2); 41,030-40,908(3); 40,110-39,925(4); 39,324-39,226(5); 39,225-39,129(6); 38,674-38,476(7); 38,335-38,274(8); 38,273-38,096(9); 37,860-37,599(10)
Hmel-ctg10	6,939-7,075(1); 7,547-7,640(2); 9,137-9,303(3); 10,186-10,276(4); 10,729-10,874(5); 11,183-11,292(6); 11,778-11,849(7); 12,102-12,222(8); 12,625-12,747(9); 13,080-13,199(10); 13,660-13,811(11); 14,221-14,330(12)
Hmel-ctg11	19,029-18,832(1); 18,425-18,268(2); 17,905-17,815(3); 17,434-17,289(4); 16,862-16,753(5); 16,364-16,281(6); 15,988-15,880(7); 15,237-15,161(8); 14,824-14,662(9); 14,077-13,891(10)
Hmel-ctg12	6,505-6,431(1); 6,324-6,208(2); 6,059-5,902(3); 4,614-4,524(4); 4,120-3,975(5); 3,674-3,565(6); 3,367-3,284(7); 2,980-2,872(8); 2,484-2,408(9); 2,294-2,132(10); 1,748-1,487(11)
Hmel-ctg13	35,582-35,366(1); 31,415-31,300(2); 30,927-30,805(3); 30,616-30,432(4); 30,101-30,018(5); 29,653-29,544(6); 29,012-28,927(7); 28,542-28,427(8); 28,179-28,057(9); 27,369-27,250(10); 26,874-26,646(11)
Hmel-ctg14	22,435-22,281(1); 14,853-14,760(2); 13,987-13,865(3); 12,429-12,275(4); 11,927-11,811(5); 11,359-11,250(6); 11,010-10,949(7); 10,570-10,450(8); 10,449-10,354(9); 10,118-9,938(10); 9,710-9,365(11)
Hmel-ctg15	4,680-4,770(1); 6,055-6185(2); 6,537-6,624(3); 7,364-7,400(4); 7,401-7,436(5); 7,953-8,043(6); 8,375-8,520(7); 8,968-9,077(8); 9,326-9,409(9); 9,713-9,821(10); 10,377-10555(11); 10,879-10,949(12); 11,385-11,766(13)
Hmel-ctg16	1,347-1,437(1); 2,478-2,608(2); 3,202-3,289(3); 3,651-3,723(4); 3,810-3,900(5); 4,433-4,578(6); 4,820-4,929(7); 5,217-5,300(8); 5,773-5,881(9); 6,384-6,564(10); 7,185-7,255(11); 7,808-7,901(12)
Hmel-ctg17	22,691-22,537(1); 22,347-22,254(2); 21,239-21,117(3); 20,630-20,476(4); 19,987-19,871(5); 19,010-18,901(6); 18,249-18,188(7); 17,410-17,194(8); 16,506-16,326(9); 15,947-15,590(10)
Hmel-ctg18	8,692-8,616(1); 8,430-8,340(2); 7,291-7,169(3); 6,837-6,683(4); 6,575-6,459(5); 5,829-5,720(6); 5,496-5,432(7); 4,764-4,629(8); 4,628-4,548(9); 4,088-3,908(10); 3,236-1,437(11)
Hmel-ctg19	168-284(2); 840-959(3); 1,932-2,057(4); 2,779-2,924(5); 3,192-3,304(6); 3,675-3,867(7); 4,244-4,390(8); 5,020-5,109(9); 5,185-5,353(10)

Supplementary Table 3 (4/4)

Sequence name	Position of exons (exon #)
Hmel-ctg20	295-417(3); 964-1,089(4); 1,598-1,743(5); 2,059-2,168(6); 2,646-2,723(7); 3,057-3,180(8); 3,364-3,486(9); 4,062-4,181(10); 4,327-4,456(11); 4,679-4,825(12)
Hmel-ctg21	6,308-6,111(1); 4,588-4,337(2); 3,914-3,769(3); 3,508-3,399(4); 3,226-3,143(5); 2,602-2,494(6); 2,196-2,080(7); 1,952-1,833(8); 1,448-1,289(9); 1,115-1,014(10)
Hmel-ctg22[1]	11,387-11,316(2); 10,465-10,343(3); 9,435-9,278(4); 8,453-8,337(5); 7,821-7,712(6); 6,992-6,931(7); 6,500-6,351(8); 6,350-6,290(9); 5,900-5,720(10); 4,497-4,083(11)
Hmel-ctg23	15,636-15,483; 15,208-15,107; 11,801-11,609; 11,156-11,050; 10,645-10,513; 10,380-10,119 (exon 5 - exon 10)
Hmel-ctg24	625-733; 2,136-2,197; 2,95-3,166; 3,878-4,058; 4,465-4,810
Hmel-ctg25	1,060-1,212; 3,036-3,135; 4,015-4,074; 4,426-4,558; 4,813-4,920; 5,351-5,482
Hmel-ctg26	7,942-8,040; 8,905-9,021; 9,579-9,698; 10,384-10,509; 11,230-11,375
Hmel-ctg27	14,107-14,343; 15,079-15,238
Hmel-ctg28	3-116; 284-400; 1,314-1,436; 1,837-1,962
Hmel-ctg29	295-417; 959-1,084; 1,366-1,511; 1,829-1,938; 2,419-2,496

Supplementary Table 4 (1/4)

Whole genome shotgun sequence accession numbers, position, length and name attributed to *D. plexippus* gene contigs (Dpl) used to reconstruct the FAR phylogeny

Sequence name	Scaffold number (DBsource)	Full-length (Complete/Partial)	Scaffold Position (orientation)	Deduced protein length	Total number of Exons[7]	Acc. number[1]
Dpl-ctg1	AGBW01005184[2]	Complete	3,311-10,856 (+)	525	11	_
Dpl-ctg2	AGBW01011881	Complete	1,262-5,938 (+)	443	10	_
Dpl-ctg3	AGBW01011881	Complete	11,030-8,442 (-)	444	10	EHJ64478
Dpl-ctg4	AGBW01005720	Complete	10,019-12,872 (-)	466	9	_
Dpl-ctg5	AGBW01000239	Complete	56,901-59,768 (+)	519	7	_
Dpl-ctg6	AGBW01006606	Complete	4,363-159 (-)	466	11	EHJ69811[+]
Dpl-ctg7	AGBW01009292	Complete	7,508-16,267 (+)	537	10	EHJ67179[p+]
Dpl-ctg8	AGBW01004968	Complete	15,809-10,524 (-)	467	10	EHJ72231[p+]
Dpl-ctg9	AGBW01006048	Complete	4,902-18,914 (+)	512	13	EHJ70557[p+]
Dpl-ctg10	AGBW01006661	Complete	1,884-5,709 (+)	416	9	_
Dpl-ctg11	AGBW01002815	Complete	20,384-15,502 (-)	478	12	EHJ76198
Dpl-ctg12	AGBW01003594	Complete	27,207-31,517 (+)	510	11	_
Dpl-ctg13	AGBW01002291	Complete	362-5,037 (+)[8]	516	nd	Dpl_EST_ctg[4] +EHJ76979[p]
Dpl-ctg14	AGBW01013701	Partial	10,289-227 (-)	447 [6]	nd	_
Dpl-ctg15	AGBW01006041	Partial	4,697-207 (-)	480 [6]	nd	_
Dpl-ctg16	AGBW01006040[2]	Partial	3,029-21,591 (+)	405 [6]	nd	_
Dpl-ctg17	AGBW01010793	Partial	512-4,306 (+)	238 [6]	nd	EHJ65352[p]
Dpl-ctg18	AGBW01005297	Partial	23,245-24,844 (+)	159 [6]	nd	_
Dpl-ctg19	AGBW01004582	Truncated[3]	726-10,257 (+)	nd	nd	EHJ67859[p+]

Supplementary Table 4 (2/4)

Sequence name	Scaffold number (DBsource)	Full-length (Complete/Partial)	Scaffold Position (orientation)	Deduced protein length	Total number of Exons[7]	Acc. number[1]
Dpl-ctg20	AGBW01006048	Truncated[3]	92-2,687 (+)	nd	nd	EHJ70556[p]
Dpl-ctg21	AGBW01006929	Truncated[3]	20,031-17,926 (-)	nd	nd	EHJ69444[p]
Dpl-FAR1[5]	AGBW01008852	Partial	nd	214 [6]	nd	EHJ67179[p]
Dpl-FAR3[5]	AGBW01005008	Complete	nd	498	nd	EHJ72165
Dpl-FAR4[5]	AGBW01006243	Partial	nd	177 [6]	nd	EHJ70258[p]
Dpl-FAR5[5]	AGBW01004967	Complete	nd	560	nd	EHJ72233
Dpl-FAR6[5]	AGBW01002641	Complete	nd	516	nd	EHJ76493

Supplementary Table 4 (3/4)

Sequence name	Position of exons (exon #)
Dpl-ctg1	3,311-3424(1); 3,564-3,680(2); 4,683-4,805(3); 5,870-5,995(4); 6,930-7,083(5); 7,406-7,507(6); 8,329-8,521(7); 9,090-9,196(8); 9,519-9,651(9); 10,287-10,544(10); 10,709-10,856(11)
Dpl-ctg2	1,262-1,351(1); 1,444-1,560(2); 1,653-1,775(3); 1,970-2,095(4); 3,545-3,698(5); 4,024-4,125(6); 4,200-4,386(7); 4,605-4,678(8); 5,381-5,510(9); 5,713-5,938(10)
Dpl-ctg3	11,030-10,956(1); 10,882-10,766(2); 10,667-10,545(3); 10,260-10,135(4); 9,993-9,849(5); 9,701-9,600(6); 9,517-9,331(7); 9,204-9,098(8); 8,905-8,776(9); 8,661-8,442(10)
Dpl-ctg4	19,019-18,759(1); 18,596-18,351(2); 17,797-17,654(3); 17,358-17,259(4); 15,766-15,707(5); 14,946-14,814(6);13,825-13,718(7); 13,358-13,098(8);12,950-12,872(9)
Dpl-ctg5	56,901-57,095(1); 57,347-57,655(2);57,870-58,065(3); 58,335-58,533(4); 58,796-59,035(5); 59,108-59,365(6); 59,609-59,768(7)
Dpl-ctg6	4,363-4,166(1); 4,079-3,922(2); 3,762-3,672(3); 3,504-3,359(4); 2,141-2,032(5); 1,792-1,709(6); 1,547-1,439(7); 1,266-1,190(8); 974-743(9); 663-5139(10); 269-159(11)
Dpl-ctg7	7,508-7,677(1); 10,801-10,891(2); 10,973-11,095(3); 12,526-12,650(4); 12,734-12,892(5); 13,222-13,331(6); 13,607-13,674(7); 14,227-14,422(8); 15,279-15,459(9); 15,880-16,267(10)
Dpl-ctg8	15,809-15,739(1); 15,332-15,218(2); 13,025-12,909(3); 12,709-12,590(4); 12,503-12,378(5); 12,108-11,853(6); 11,657-11,471(7); 11,301-11,195(8); 10.905-10.812(9); 10,731-10,524(10)
Dpl-ctg9	4,902-4,992(1); 8,784-8,914(2); 9,356-9,443(3); 9,722-9,794(4); 10,869-10,959(5); 11,479-11,624(6); 11,997-12,106(7); 14,848-14,919(8); 15,489-15,597(9); 15,950-16,127(10); 16,631-16,701(11); 18,313-18,570(12); 18,809-18,914(13)
Dpl-ctg10	1,884-2,078(1); 2,478-2,729(2); 2,980-3,125(3); 3,289-3,398(4); 3,634-3,717(5); 4,062-4,170(6); 4,437-4,553(7); 4,701-4,820(8); 5,595-5,709(9)
Dpl-ctg11	20,384-20,187(1); 20,103-19,946(2); 19,758-19,668(3); 19,470-19,325(4); 18,530-18,421(5); 18,230-18,163(6); 17,627-17,557(7); 17,464-17,388(8); 16,873-16,711(9); 16,635-16,485(10); 16,249-16,143(11); 15,595-15,502(12)
Dpl-ctg12	27,207-27,401(1); 27,801-28,052(2); 28,308-28,453(3); 28,617-28,726(4); 29,066-29,149(5); 29,517-29,625(6); 29,892-30,008(7); 30,155-30,274(8); 30,770-30,929(9); 31,056-31,153(10); 31,379-31,517(11)
Dpl-ctg13	(exon positions from aa 128 to 516): 362-452; 727-872; 1,788-1,897; 2,159-2,242; 2,855-2,963; 3,054-3,231; 3,863-3,933; 4,213-4,470; 4,917-5,037
Dpl-ctg14	10,289-10,159(2); 9,743-9,656(3); 9,379-9,307(4); 8,249-8,159(5); 7,632-7,487(6); 7,114-7,005(7); 4,337-4,254(8); 3,676-3,568(9); 2,851-2,674(10); 2,170-2,100(11); 488-227(12)
Dpl-ctg15	4,697-4,607(2); 4,525-4,403(3); 3,837-3,713(4); 3,629-3,471(5); 3,136-3,027(6); 2,752-2,685(7); 2,136-1,941(8); 1,195-1,015(9); 594-207(10)
Dpl-ctg16	3,029-3,180(1); 14,168-14,261(2); 17,666-17,788(3); 17,902-18,056(4); 19,542-19,658(5); 20,283-20,392(6); 20,521-20,582(7); 20,717-20,933(8); 21,407-21,591(9)
Dpl-ctg17	512-687(1); 1,031-1,121(2); 2,680-2,825(3); 3,021-3,130(4); 3,456-3,539(5); 4,198-4,306(6)
Dpl-ctg18	23,245-23,433(1); 23,899-24,055(2); 24,402-24,492(3); 24,806-24,844(4)
Dpl-ctg19	726-942(1); 5,551-5,666(2); 5,898-6,020(3); missing exons; 7,839-7,954(4); 8,332-8,454(5); 8,605-8,724(6); 9,073-9,263(7); 9,769-9,835(8); 10,155-10,257(9)

Supplementary Table 4 (4/4)

Sequence name	Position of exons (exon #)
Dpl-ctg20	92-153(1); 244-421(2); 1,053-1,123(3); 1,407-1,664(4); 2,110-2,215(5); 2,469-2,687(6)
Dpl-ctg21	20,031-19,894(1); 19,460-19,350(2); 19,125-19,003(3); 18,051-17,926(4)
Dpl-FAR1[5]	-
Dpl-FAR3[5]	-
Dpl-FAR4[5]	-
Dpl-FAR5[5]	-
Dpl-FAR6[5]	-

[1] Monarch genome project Assembly DanPle_1.0, [P] Indicates that the genome project prediction is partial, [+] refers to a gene prediction that was corrected in this study

[2] Alternative scaffolds for Dpl-ctg1: AGBW01010887 (ex1-x7), Dpl-ctg16: AGBW01011560 (ex3-ex7)

[3] Missense mutations in predicted coding exons or missing internal exons or partial genes that do not fall over scaffold boundaries

[4] Combination of the following ESTs: Acc. Nrs. EY269663, EY270061, EY257734

[5] FAR-like genes predicted from the Monarch genome project; Dpl-FAR2 has Acc. Nr. EHJ67859 and corresponds to Dpl-ctg19

[6] Partial deduced aa sequences

[7] Only assigned for full-length FAR-like proteins manually curated in this study, nd = not determined for partial sequences

[8] Corresponds to aa residues 128 to 516 from AGBW01002291, the first 127 aa residues were assembled from info from [4].

Supplementary Table 5

List of species names and corresponding published accession numbers for sequences[a] used in the FAR phylogeny

Gene abbreviation	Species Name	Accession number
Aip_pgFAR	*Agrotis ipsilon*	JX679210
Ban_EST1	*Bicyclus anynana*	GE734925
Ban_EST2	*Bicyclus anynana*	GE724246
Ban_EST3	*Bicyclus anynana*	GE719384+ GE719381+ GE719382
Ban_EST4	*Bicyclus anynana*	GE720025+ GE720022
Ban_EST5	*Bicyclus anynana*	BAP05235
Ban_EST6	*Bicyclus anynana*	GE719150+ GE719145
Ban_EST7	*Bicyclus anynana*	GE730527+ GE730530
Bmo_pgFAR	*Bombyx mori*	BAC79426
Her_EST1	*Heliconius erato*	HEP03568
Her_EST2	*Heliconius erato*	HEP04011
Har_pgFAR	*Helicoverpa armigera*	JF709978
Has_pgFAR	*Helicoverpa assulta*	JF709977
Hsu_pgFAR	*Heliothis subflexa*	JF709976
Hvi_pgFAR	*Helliothis virescens*	EZ407233
Ofu_pgFAR	*Ostrinia furnacalis*	AGG19591
Ola_pgFAR	*Ostrinia latipennis*	AGG19592
OnuE_pgFAR	*Ostrinia nubilalis E*	FJ807735
OnuZ_pgFAR	*Ostrinia nubilalis Z*	FJ807736
OnuZ1	*Ostrinia nubilalis Z*	GU808250
OnuZ2	*Ostrinia nubilalis Z*	GU808251
OnuZ3	*Ostrinia nubilalis Z*	GU808252
OnuZ4	*Ostrinia nubilalis Z*	GU808253
OnuZ5	*Ostrinia nubilalis Z*	GU808254
OnuZ6	*Ostrinia nubilalis Z*	GU808255
Opa_pgFAR	*Ostrinia palustralis*	AGG19593
OscE_pgFAR	*Ostrinia scapulalis E*	AGG19594
OscZ_pgFAR	*Ostrinia scapulalis Z*	AGG19595
Oze_pgFAR	*Ostrinia zealis*	AGG19598
Oza_pgFAR	*Ostrinia zaguliaevi*	AGG19597
Onr.za_pgFAR	*Ostrinia nr zaguliaevi* JML-2013	AGG19596
Yev_pgFAR	*Yponomeuta evonymella*	GQ907232
Yev_FARI	*Yponomeuta evonymella*	GQ907231
Yev_FARIII	*Yponomeuta evonymella*	GQ907233
Ypa_pgFAR	*Yponomeuta padella*	GQ907235
Yro_pgFAR	*Yponomeuta rorrella*	GQ907234

[a]except for accession numbers from *Bombyx mori* (swdb-ctgs) that are listed in[3] , as well as the in silico predictions for *Heliconius melpomene* (Hmel-ctgs) and *Danaus plexippus* (Dpl-ctgs) that are listed in Supplementary Tables 3 and 4, respectively.

Supplementary Table 6

List of oligonucleotide primers

Primer name	Primer sequence (5'-3')	amplicon size (bp)
5´and 3´ cDNA RACE (5R or 3R)		
Ban-Δ11-5R	TGTTGCCCCAAAGATGAGCAGCGCTGTT	\
Ban-Δ11-5Rnested	TCATGTGGAGGTTGAAGACGTAGCGCATG	
Ban-Δ11-3R	CGTCGGGGCAAGTTCATCGACATGTCC	\
Ban-Δ11-3Rnested	CTATGCCAACCCGGTGCTAAGGTTCCAG	
Ban-wFAR1-5R	CTCCTTGTTTCTCCCGCACCAAGAG	\
Ban-wFAR1-5Rnested	CTTTCTCTATTCCGGGACAACTGTACAG	
Ban-wFAR1-3R	CACAACTCGGTATCAAGGCGGAGGATG	\
Ban-wFAR1+2-3Rnested[a]	ACAGGCCATGAACGTAAACGTAGCGGG	
Ban-wFAR1-3R-Int-F[b]	ATTTTACGATGTGCCGATGCCAATGG	\
Ban-wFAR1-3R-Int-R[b]	CGTCACAAACAAACCGTATTCGGCTCAC	
Ban-wFAR2-5R	CGACACCGGTGCTGGATACAAAACTTCC	\
Ban-wFAR2-5Rnested	CGCCGTGGAAACATATACGAAGGCCTTA	
RT-PCR		
Ban-Δ11s[c]	CGTCGGGGCAAGTTCATCGACATGTCC	235
Ban-Δ11as[c]	TGTTGCCCCAAAGATGAGCAGCGCTGTT	
Ban-wFAR1s	ATCATGGACCAGGCCATAGCGGAATG	336
Ban-wFAR1as[d]	GTGGTATACTATCGATACTTTTTCAGCCAGG	
Ban-wFAR2s	AAGATGGATCGAAATGTTAACAACAATT	441
Ban-wFAR2as[d]	GTGATAAACGAATGATACCTCCTTAATCAGA	
16SRNA-s	TGAAGGGCTGCAGTATTTTG	397
16SRNA-as	TCGAGGTCGCAAACTCTTTT	
ORF amplification for functional assay		
Ban-Δ11-s[e]	*GCGGATCC*aaaATGgCGCCCTCCGCAA	990
Ban-Δ11-as[e]	GC*GAATTC*TCACTCAGTCTTATCTCGAAAGCCCCA	
Ban-wFAR1-s[e]	atcATGgACCAGGCCATAGCGGAATG	1364
Ban-wFAR1-as[e]	CACGGTGAGTTATATTCTATACGGAATTA	
Ban-wFAR2-s[e]	aagATGgATCGAAATGTTAACAACAATT	1440
Ban-wFAR2-as[e]	TTAAGATTTACACAAAAACTGCCTTATTC	

[a] Primer that led to amplification of both Ban-wFAR1 and Ban-wFAR2 3' cDNA ends

[b]Internal primer pair designed to sequence the Ban-wFAR1 long 3'UTR cDNA.

[c]Primer pair encompassing the junction of intron III of desaturases.

[d]Reverse primers overlapping the junction between exon4 and exon5.

[e,f,g]The start or stop codon is underlined except when primer is designed on 3'UTR, the kozak sequence is indicated in lower case letters and restriction sites are in italics.

Supplementary references

1 Dallerac, R. *et al.* A Δ9 desaturase gene with a different substrate specificity is responsible for the cuticular diene hydrocarbon polymorphism in *Drosophila melanogaster*. *Proc. Natl. Acad. Sci. USA* **97**, 9449-9454 (2000).

2 Rosenfield, C.-L., You, K. M., Herrick-Marsella, P., Roelofs, W. L. & Knipple, D. C. Structural and functional conservation and divergence among acyl-CoA desaturases of two noctuid species, the corn earworm, *Helicoverpa zea*, and the cabbage looper, *Trichoplusia ni*. *Insect Biochem. Mol. Biol.* **31**, 949-964 (2001).

3 Liénard, M. A., Hagström, Å. K., Lassance, J.-M. & Löfstedt, C. Evolution of multi-component pheromone signals in small ermine moths involves a single fatty-acyl reductase gene. *Proc. Natl. Acad. Sci. USA* **107**, 10955-10960 (2010).
